# Comparative transcriptomic analysis of germinating rice seedlings to individual and combined anaerobic and cold stress

**DOI:** 10.1186/s12864-023-09262-z

**Published:** 2023-04-06

**Authors:** Ranjita Thapa, Rodante E. Tabien, Charles D. Johnson, Endang M. Septiningsih

**Affiliations:** 1 Department of Soil and Crop Sciences, Texas A&M University, College Station, TX 77843 USA; 2 Present address: Section of Plant Breeding and Genetics, School of Integrative Plant Sciences, Cornell University, Ithaca, NY 14853 USA; 3 Texas A&M AgriLife Research Center, Beaumont, TX 77713 USA; 4 Genomics and Bioinformatics Service, Texas A&M AgriLife Research, College Station, TX 77843 USA

**Keywords:** Rice (*Oryza sativa* L.), Anaerobic germination (AG) stress, Cold stress during germination, Combined stress, RNA-seq, Differentially expressed genes (DEGs), Hub genes

## Abstract

**Background:**

Rice is one of the most important cereals consumed worldwide. Two major abiotic factors affecting rice plants in different growth stages are flooding stress and cold stress. These abiotic stresses can take place independently or simultaneously and significantly affect rice plants during germination and seedling growth. Fortunately, a wide array of phenotypic responses conferring flooding stress and chilling stress tolerance exist within the rice germplasm, indicating the presence of different molecular mechanisms underlying tolerance to these stresses. Understanding these differences may assist in developing improved rice cultivars having higher tolerance to both stresses. In this study, we conducted a comparative global gene expression analysis of two rice genotypes with contrasting phenotypes under cold stress, anaerobic stress, and combined cold and anaerobic stress during germination.

**Results:**

The differential gene expression analysis revealed that 5571 differentially expressed genes (DEGs), 7206 DEGs, and 13279 DEGs were identified under anaerobic stress, cold stress, and combined stress, respectively. Genes involved in the carbohydrate metabolic process, glucosyltransferase activity, regulation of nitrogen compound metabolic process, protein metabolic process, lipid metabolic process, cellular nitrogen compound biosynthetic process, lipid biosynthetic process, and a microtubule-based process were enriched across all stresses. Notably, the common Gene Ontology (GO) analysis identified three hub genes, namely Os08g0176800 (similar to mRNA-associated protein mrnp 41), Os11g0454200 (dehydrin), and OS10g0505900 (expressed protein).

**Conclusion:**

A large number of differentially expressed genes were identified under anaerobic, cold conditions during germination and the combination of the two stress conditions in rice. These results will assist in the identification of promising candidate genes for possible manipulation toward rice crops that are more tolerant under flooding and cold during germination, both independently and concurrently.

**Supplementary Information:**

The online version contains supplementary material available at 10.1186/s12864-023-09262-z.

## Background

Rice is a staple food crop for more than half of the world’s population. Many rice-growing regions, especially in South and Southeast Asia, are in flood-prone lowland areas [1]. Flash flooding due to unpredicted rainfall is a common phenomenon in such areas, severely affecting rice crops in various stages of their development, requiring different strategies to address the problems [2–7]. Rice is adapted to aquatic ecology; therefore, it has a unique mechanism of germinating and developing a coleoptile underwater [8]. The coleoptile elongation is achieved by rapid elongation of basal cells (up to 200 um in 12 h) immediately after emerging from an embryo [9]. However, not all rice accessions possess vigorous growth underwater, and the germination potential varies greatly among rice genotypes under flooded conditions.

Another abiotic stress that is detrimental to rice germination and growth is cold stress. Cold stress alone or its combination with unexpected heavy rains at the beginning of rice-growing seasons are common in rice-growing areas, especially in temperate regions, including the U.S. The recommended date of rice planting in Texas is March 15 to April 15 (https://beaumont.tamu.edu/elibrary/Newsletter/2010_April_Newsletter.pdf), but the yield can be significantly improved if early planting is successful. The main challenges for early planting in Texas are cold weather and unexpected rain. These stresses cause poor emergence and poor establishment of the germinating rice seeds. Robust seedling establishment is necessary to improve yield stability under stress-prone conditions, and tolerance of anaerobic germination (AG) and cold temperature are the most desirable traits. Understanding the molecular mechanisms underlying tolerance to these stresses and subsequently using this knowledge to assist in developing rice varieties tolerant to these conditions can potentially improve rice seedling establishment, which translates to more robust and productive rice growth.

Many studies on QTL mapping and genome wide association studies (GWAS) on AG tolerance in rice have been reported [10–22]. Notably, a gene underlying one of the QTLs, *qAG-9-2*, has been cloned as *trehalose-6-phosphate-phosphatase* (*OsTPP7*), which is involved in starch mobilization during germination [23]. Previous biochemical and enzymatic experiments have demonstrated the involvement of starch breakdown mechanisms and the induction of amylase during low oxygen germination in rice [24–26]. A positive correlation between coleoptile length and total amylolytic activities has been reported [27]. Under anoxic conditions, the level of ethanol is elevated in the fast-growing coleoptiles, which suggests that energy is generated from the fermentative metabolism pathway [28–30]. Recent studies have also demonstrated that maintaining a high rate of energy production as well as fluxes between the glycolytic and fermentative pathways is crucial for survival during anaerobic conditions [31, 32].

A few studies on genome-wide transcriptome analysis to investigate the gene expression analysis of rice coleoptiles under hypoxic or anoxic conditions have been reported. The results of the studies showed that some common molecular mechanisms are involved in coleoptile growth, including carbohydrate metabolism, fermentation, hormone induction, and cell division and expression [33–35]. A study on the tip and basal segments of oxygen-deprived coleoptiles of rice showed that the gene expression is region-specific. Furthermore, the metabolic activities are different along the length of the coleoptile under normoxic (air), hypoxic (3%), and anoxic conditions [9]. Tolerant plants have developed the ability to generate ATP in the absence of oxygen through fermentative metabolism and to develop specific morphologies (such as air channels and enhanced shoot elongation) that improve the entrance of oxygen [29, 36–43]. Moreover, gene expression studies on plants exposed to low oxygen conditions revealed the up-regulation of genes coding for signal transduction components [44], transcription factors [45, 46], non-symbiotic hemoglobin [47], ethylene biosynthesis [48], nitrogen metabolism [49], and cell wall loosening [50].

Similarly, several studies on the identification of cold tolerance QTLs and genes during germination and seedling stages using biparental QTL mapping and GWAS have been reported [51–53]. A QTL for cold tolerance, *COLD1*, was identified on the long arm of chromosome 4. The causal gene encodes a regulator of G-protein signaling, which together with the G-protein α subunit plays a key role in activating the Ca2+ channel for sensing low temperature and accelerating G-protein GTPase activity, contributing to the adaptation of *japonica* rice to chilling conditions [54]. Similarly, it was reported that the transcription factor bZIP73 may have contributed to *japonica* adaptation to cold climates [55]. A gene underlying a QTL for cold tolerance at the booting stage, *CTBa*, encoding a conserved leucine-rich repeat, has also been cloned [56].

Several transcriptomic studies to investigate the underlying mechanisms of cold tolerance in rice using microarray technologies have been reported [57–61]. These studies revealed that a large number of cold-responsive genes could be categorized into three groups. The first group is the signaling components that regulate gene expressions in cold stress responses, which include protein kinases and transcription factors. Secondly, the functional components that directly protect plant cells against cold stress. These include enzymes in metabolic pathways, aquaporins and proteins involved in photosynthesis. Lastly, the small noncoding RNAs such as the microRNAs (miRNAs).

A comprehensive understanding of the molecular mechanisms of anaerobic stress and cold adaptation is an important area of research. The tolerance or sensitivity to these abiotic factors is a complex phenomenon involving responses at the molecular, biochemical, and physiological levels. However, limited research has been performed in comparative genome-wide transcriptomics analysis across these stresses, especially when they occur together. The goal of this study was to evaluate the differentially expressed genes in two contrasting rice cultivars in response to anaerobic, cold, and a combination of anaerobic and cold stress during germination to better understand the underlying molecular mechanisms and metabolic pathways that contribute to tolerance.

## Results and discussion

### Overview of cDNA library sequencing

Tissues from four replicates per sample were harvested to construct cDNA libraries of germinating rice seeds from two contrasting genotypes, Darij (tolerant to anaerobic germination (AG) and cold stress conditions) and line 4610 (susceptible), grown under control, AG stress, cold stress, and combined cold and AG stress conditions. The cDNA libraries were then sequenced with the HiSeq 4000 using 2X75 [62]. An average of 17.62, 23.00, and 20.53 million clean reads were generated from AG stress, cold stress, and combined AG and cold stress and mapped to the Nipponbare reference genome with Hisat2 [63], with 91.62% to 96.78% clean reads from all samples successfully mapped to the reference genome (Supplementary Tables 1, 2, 3, 4 and 5). The total gene counts were normalized using the DESeq2 package, and the expression signals of each gene were calculated. The PCA analysis based on regularized log transformation of total gene counts with DESeq2 showed 58%, 77%, and 59% variance explained by PC1 under AG, cold and combined AG and cold stress, respectively, and 34%, 16%, and 35% variance explained by PC2 under AG, cold and combined AG and cold stress, respectively (Supplementary Figure 1).

### Phenotypic evaluation

Based on the phenotypic evaluation, the two selected lines showed contrasting differences under AG stress, cold stress and combined AG and cold stress (Table 1). Under control conditions, the mean coleoptile length of Darij was 1.54 cm and 4610 was 1.2 cm at 4 days after sowing (DAS) and 2.48 cm and 3 cm, respectively, at 7 DAS. There was no statistically significant difference (*P* value < 0.05) in the coleoptile length between these two lines under control conditions. In contrast, a statistically significant difference (*P* value < 0.05) was observed among these lines under stress treatments. Under AG stress condition, the mean coleoptile length of Darij was 2.2 cm and 4610 was 0.25 cm at 4 DAS. Under cold conditions, the coleoptile length of Darij was 0.41 and 4610 was 0.025 cm at 21 DAS and 0.86 cm and 0.14 cm, respectively, at 28 DAS. The mean plumule length of Darij and 4610 after 4 days of recovery at 30°C was 2.63 cm and 0.63 cm, respectively. Under combined stress of AG and cold, the mean coleoptile length of Darij was 0.43 cm and 0.68 cm at 28 DAS and 35 DAS, respectively, whereas the susceptible line, 4610, did not germinate at all, even at 35 DAS. All variables (coleoptile length under AG stress, shoot length under cold exposure, plumule growth recovery, and coleoptile length under the combination of AG and cold stress) showed superior performance of the tolerant line (Darij) over the susceptible line (4610). The tolerant line was superior for shoot length under AG stress, cold stress, and the combined stress, and the seedling growth was increased in the tolerant line even after 21 days of cold exposure. Likewise, the plumule growth recovery of the tolerant line was also much better than the susceptible one. On the other hand, the seedling growth of the susceptible line was negligible under cold stress and showed no germination under combined AG and cold stress. Similarly, the tolerant line showed significantly longer coleoptile growth under the submerged condition in 4 DAS. Alpi and Beevers, 1983 [64] and Kawai and Uchimiya, 2000 [65] reported that when flooding occurs after direct seeding, tolerant lines germinate faster. At the same time, their coleoptiles grow faster, enabling them to reach the more aerated zone and oxygen referred to as the snorkel effect. The significant reduction of coleoptile growth under cold and submerged conditions during the initial stage of germination in rice is a limiting factor for good crop establishment [66, 67]. Thus, selecting genotypes with longer coleoptile lengths is critical for cold and flooding tolerance during germination [68, 69].

**Table 1:** Phenotypic data of coleoptile length of tolerant and susceptible lines under AG stress, cold stress and combination of AG and cold stress and plumule length after cold recovery

Cold stress	Tolerant (Darij)			Susceptible (4610)		
	Range (cm)	Average (cm)	Stdev (cm)	Range (cm)	Average (cm)	Stdev (cm)
Control (30°C, 7 DAS)	1.28 - 3	2.48	0.44	2.53 - 3.60	3	0.42
Cold stress (13°C, 21 DAS)	0.35 - 0.60	0.41	0.07	0 - 0.1	0.025	0.05
Cold stress (13°C, 28 DAS)	0.62 - 1.16	0.86	0.16	0.1 -0.2	0.14	0.04
Plumule recovery (30°C, 4DAS)	2.05 - 3.57	2.63	0.45	0 - 1.12	0.63	0.3
AG stress	Tolerant (Darij)			Susceptible (4610)		
	Range (cm)	Average (cm)	Stdev (cm)	Range (cm)	Average (cm)	Stdev (cm)
Control (30°C, 4 DAS)	1.42 - 1.65	1.54	0.09	1.09 - 1.3	1.2	0.08
AG stress (30°C, 4DAS)	1.93 - 2.55	2.2	0.26	0.17 - 0.33	0.25	0.07
AG + Cold stress	Tolerant			Susceptible		
	Range (cm)	Average (cm)	Stdev (cm)	Range (cm)	Average (cm)	Stdev (cm)
AG + Cold stress (13°C, 28 DAS)	0.1 - 0.8	0.43	0.17	0	0	0
AG + Cold stress (13°C, 35 DAS)	0.1 - 2.5	0.68	0.44	0	0	0

### Differential expression analysis

**Fig. 1:**
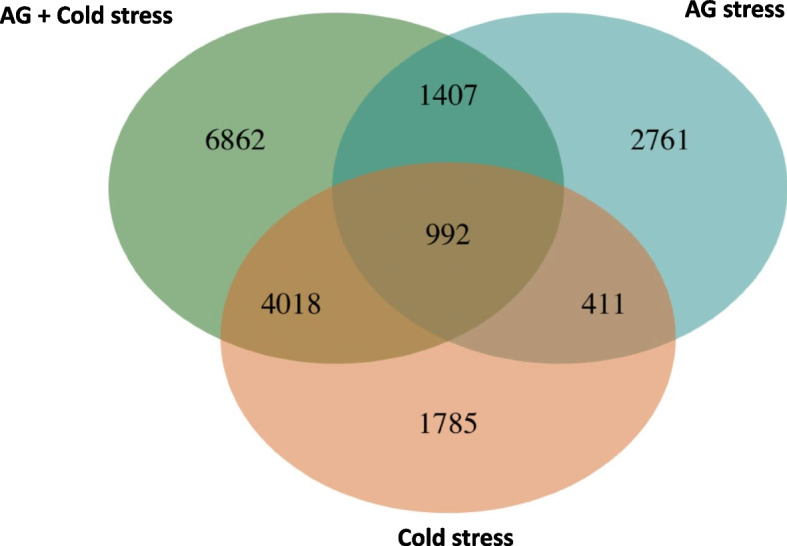
Ven diagram of DEGs across stresses. Total number of DEGs under anaerobic germination, cold germination and combination of anaerobic and cold stress during germination conditions are shown

To further examine whether the gene expression was affected by treatment only or both genotype and treatment, multi-factorial linear model testing was performed on our whole transcriptome dataset using DESeq2. Applying an FDR corrected *p*-value of < 0.05, the separate analysis of Darij and 4610 under AG stress versus control showed that the total number of differentially expressed genes (DEGs) was 16,383 in Darij and 15,435 genes in line 4610, with 9,798 DEGs in common (Fig. 1a). In this analysis, the total number of upregulated genes was 8,082 in Darij and 7,579 in 4610, while the total number of downregulated genes was 8,744 in Darij and 7,856 in 4610 (Supplementary Table 6). Under cold stress, 12,101 and 16,240 DEGs were identified in Darij and 4610, respectively, compared to the control, with 7,962 DEGs in common. The total number of upregulated genes in Darij and 4610 were 5,612 and 8,010, respectively, and the total number of downregulated genes in Darij and 4610 were 6,489 and 8,230, respectively. For the combination of AG and cold stress, 19,040 and 21,906 DEGs were identified in Darij and 4610, respectively, with 14,382 DEGs in common. The total number of upregulated genes in Darij and 4610 were 8,435 and 10,590, respectively, and the total number of downregulated genes in Darij and 4610 were 10,605 and 11,316, respectively. The total number of DEGs were higher in both lines under AG stress and under the combination of AG and cold stress. The number of DEGs was higher in the susceptible line, 4610, under cold stress and the combination of AG and cold stress. Similar findings were previously reported [70, 71]. Da Maia et al. 2016 revealed 19 times more DEGs in cold-sensitive genotype than in cold-tolerant genotype in response to cold, and Pan et al. 2020 identified more downregulated DEGs in cold-sensitive rice. This could be due to severe damage imposed on the susceptible lines at the cellular and molecular level than on the tolerant lines.

We found statistically significant interaction effects indicating that the gene expression under different stresses is regulated differently depending on the genetic background, i.e., variation of coleoptile elongation in diverse rice accessions could be potentially determined by genotype-specific gene expression. Considering both genotype and treatment effects, 5,571 DEGs were identified under AG stress where 2,294 genes were up-regulated, and 3,277 genes were down-regulated. Under cold stress, 7,206 DEGs were identified, where 3,276 genes were up-regulated, and 3,930 genes were down-regulated. Under combined stresses, 13,279 DEGs were identified with 6,282 up- and 6,997 down-regulated genes (Supplementary Table 7).

From the pairwise comparison, the highest number of common DEGs (5,010) were found between cold and combined AG and cold stress, followed by DEGs (2,399) between AG and combined AG and cold stress and DEGs (1,403) between AG stress and cold stress (Fig. 1). The results of shared differentially expressed genes indicated a greater relationship between cold stress and combined AG and cold stress, followed by AG stress and combined AG and cold stress. Among all DEGs, 992 genes were shared among all three stresses, of which 430 genes were commonly up-regulated, 195 genes were commonly down-regulated, and 367 of them were up-regulated under one condition and down-regulated under the other two conditions or inversely. Among 5,010 common DEGs between cold and combined AG and cold stress, 2,112 genes were up-regulated, 2,726 genes were down-regulated, and 172 genes were up-regulated under one condition and down-regulated under the other condition. Among 2,399 common DEGs between the AG and combined AG and cold stress, 515 genes were up-regulated, 901 genes were down-regulated, and 983 genes were up-regulated under one condition and down-regulated under the other condition. Among 1,403 common DEGs between AG and cold stress, 289 genes were up-regulated, 629 genes were down-regulated, and 485 genes were up-regulated under one condition and down-regulated under the other condition. The total number of differentially expressed genes was higher in combined cold stress and AG stress than under individual stress indicating that the combined stress could have caused severe damage to the cellular and metabolic processes compared to individual stress, leading to greater complexity of gene regulation.

### Common and unique DEGs in the tolerant and susceptible line

In broad GO annotation, we observed some similarities and differences among the tolerant and susceptible lines. The GO enrichment analysis identified a total of 309, 278 and 289 enriched GO terms in the tolerant line, Darij, and a total of 318, 291 and 297 enriched GO terms in the susceptible line, 4610, under AG stress, cold stress and the combination of AG and cold stress, respectively (Supplementary Table 8). There were 247, 220 and 242 common GO terms between the tolerant line and susceptible line under AG stress, cold stress and combination of AG and cold stress, respectively. In the tolerant line, a total of 62, 58 and 47 unique GO were detected under AG stress, cold stress, and a combination of AG and cold stress, respectively. In the susceptible line, a total of 71, 71 and 55 unique GO terms were identified under AG stress, cold stress, and a combination of AG and cold stress, respectively. Under AG stress, the common GO terms included the carbohydrate metabolic process, the nitrogen compound metabolic process, the carboxylic acid metabolic process, the glucose metabolic process, glycolysis, thylakoids, and photosynthesis. On the other hand, the unique GO terms in the tolerant line included the cellular aromatic compound metabolic process, the aromatic compound biosynthetic process, ATPase activity, transferase activity and response to abiotic stimulus; while the unique GO terms in the susceptible line included the microtubule-based process, GTPase activity, the regulation of signaling process, cytoskeletal and peroxidase activity. The molecular functions related to 6-phosphofructokinase activity, ATPase activity, acetyltransferase activity, and transferase activity were uniquely enriched in the tolerant line, while response to oxidative stress, the regulation of signaling process, and GTPase regulator activity were uniquely enriched in susceptible line.

During flooded conditions, rice plants are threatened by a reduction in oxygen availability either due to slower diffusion of oxygen through water or continuing competition for oxygen with respiring microorganisms [36, 72]. Major complications associated with low oxygen stress are related to energy crises caused by the inhibition of mitochondrial oxidative phosphorylation and subsequent reduction in ATP production. The shortage of ATP causes impaired functioning of the plasma membrane H^+^ pumping ATPase, which leads to hypoxia-induced acidification of cytoplasm [73]. The adaptability of plants to low oxygen stress is generally considered in terms of anaerobic carbon metabolism [74]. The enrichment of the cellular nitrogen metabolic process and regulation of the nitrogen metabolic process in this study show the role of anaerobic carbon metabolism in AG stress conditions. Several researchers have also reported the contribution of nitrogen metabolism in cellular acclimation to low oxygen stress in plants [75, 76]. Low oxygen stress significantly affects several nitrogen metabolism processes, including nitrogen absorption, nitrate and nitrite reduction, nitric oxide (NO) production, ammonium assimilation, and amino acid metabolism [74]. Several studies have shown that nitrate fertilization improves hypoxia tolerance and limits hypoxia stress in the roots [77–79]. The higher number of genes involved in the cellular nitrogen compound metabolic process, NAD or NADH binding, oxidoreductase activity, and glucosyltransferase activity might contribute to tolerance. O-glucosyltransferase 2 was previously shown to be involved in *Arabidopsis* seed germination [80]. Fructokinase (OsFK2) was previously reported to be induced by anoxia [81]. Fructokinase plays a key role in driving the hexose (fructose and UDP-glucose) released by sucrose synthase into glycolysis which helps in increasing tolerance under anaerobic conditions; this might be the reason for increased 6-phosphofructokinase activity in the tolerant line, Darij.

The biological processes related to cellular lipid metabolic process, lipid biosynthetic process, and molecular function related to lipid binding were also uniquely enriched in the tolerant line. The number of genes enriched in lipid localization and lipid transport were higher in the tolerant line than the susceptible line. The hydrogenation of unsaturated fatty acids and the biosynthesis of lipids were hypothesized to be adaptive mechanisms under anaerobic stress because they function as terminal acceptors of respiratory electrons and protons [82, 83] and lipid biosynthesis and accumulation were found to bypass the major problem of anaerobiosis in *Echinochloa phyllopogon*[82]. However, the rice coleoptile requires anaerobic synthesis of lipids. This synthesis is an adaptive mechanism to achieve the turnover of saturated fatty acids, phospho-, glyco- and neutral lipids under extreme conditions of oxygen deficiency, thereby stabilizing cell membranes under strict anoxia [84].

The enzyme scavengers, and peroxidase activity enriched exclusively in susceptible line 4610, suggests that reactive oxygen species (ROS) metabolism was higher in this line. Additionally, the metabolism carried out by mitochondria is directly connected to oxygen availability. Anoxia-germinated seedlings of rice develop coleoptiles with intact structure and functional activity of mitochondria capable of oxidative phosphorylation [8]. The GO process involving the cellular component for the mitochondrial membrane is also exclusively enriched in the susceptible line. This may suggest that there were higher activities of the genes related to mitochondria in susceptible genotypes. Oxygen deprivation might have affected mitochondrial ultrastructure leading to susceptibility of genotypes under submerged conditions.

The genes involved in photosynthesis were found to be downregulated under AG stress in both lines; however, the number of downregulated genes in the susceptible line was higher. This agrees with the hypothesis that photosynthetic ability is affected under stresses, including flooding stress [85]. Interestingly, several researchers have reported the upregulation of photosynthesis-related genes under flooding. Nanjo et al. (2011) reported the upregulation of photosynthesis-related genes after imposing flooding at 2 d old seedling stage of soybean [86]. The upregulation of photosynthetic-related genes in *Arabidopsis* after imposing low oxygen stress for 3h and in poplar after imposing flooding stress for 5 h was also reported [87]. However, Lee et al. (2014) reported the downregulation of photosynthesis-related genes in the leaves of rape seedlings after 3 d of water-logged conditions [88]. This suggests that flood stress stimulates the expression of photosynthesis-related genes during initial stress response and represses the expression of these genes at later stages. Reduced photosynthesis during flooding stress could be due to the production of ROS [89]. High levels of ROS can damage cells through peroxidation of lipids, oxidation of proteins, and other pathways, finally leading to cell death [90, 91]. Moreover, the number of enriched genes in the biological function of heme binding is higher in the tolerant line than in the susceptible line. Non-symbiotic hemoglobins induced under hypoxia were reported to play a role in modulating low oxygen responses as low oxygen concentration induces class-1 hemoglobin gene GLB1 in Arabidopsis [89, 92] which was identified to increase survival under severe hypoxia [93]. Also, non-symbiotic hemoglobins may act as NO-detoxifying agents under hypoxia, where conspicuous amounts of NO are generated [94].

Under cold stress, some of the common GO categories were carbohydrate metabolic process, nitrogen compound metabolic process, lipid metabolic process, carboxylic acid biosynthetic process, cytoskeletal, and microtubule-associated complex. GO terms such as glucan metabolic process, lipid localization, phospholipid metabolic process, mitochondrial part, ribosome were found to be unique in the tolerant line, while GO terms such as glycolysis, chromosome organization, ATPase activity, heat shock protein binding, and cellular response to stress were found to be unique in the susceptible line. All the common GO terms have a larger number of enriched genes in the susceptible line. High expression of cellulose synthase genes was reported to be important for tolerance to biotic [95] and abiotic stresses in rice plants [96]. Microtubule organization for cellulose synthase positioning and cellulose deposition has been demonstrated to be essential for biomass production during salt stress in *Arabidopsis* [97]. In this study, we identified microtubule-based process, microtubule cytoskeleton, microtubule-associated complex, and microtubule motor activity in both tolerant and susceptible lines. However, the number of enriched genes in the susceptible line was higher than in the tolerant line. Additionally, we identified enrichment of genes related to lipid localization, lipid transport, and phospholipid metabolic process exclusively in the tolerant line, which might partly be the reason for higher tolerance. Cell membranes are the major target for temperature susceptibility in plants [98], and a strong association between temperature and fatty acid content of plant membranes has been demonstrated [99]. An increasing level of saturated fatty acids has been demonstrated to be correlated with higher sensitivity to cold temperatures in *Arabidopsis* plants [100], and unsaturated fatty acids, due to their lower melting point, were reported to maintain membrane fluidity [99].

We also identified a uniquely enriched GO related to cellular component, clathrin coat, in the tolerant line, which was previously reported to play an important role in Ca^2+^ signaling pathways across the plasma membranes [101]. Q7XKE9 (Clathrin light chain 1) protein was found to be a differentially expressed protein in cold tolerant lines [102]. Chilling stress manifests in a decrease of photosynthesis, an increase of reactive oxygen species (ROS), over-reduction of chloroplast electron transport chain, and damage to membrane integrity which causes cellular dehydration, osmotic imbalance, and other deleterious effects [103, 104]. Our study shows that the tolerant line has more genes expressed for photosynthesis and maintenance of structural membrane integrity. Previous studies have shown that circadian rhythms influence photosynthetic processes and play a vital role in coordinating photosynthetic activity with the diurnal changes in light. The studies have also demonstrated that chilling temperatures disrupt photosynthetic processes in warm-climate plants [105–108]. Our results confirm previous findings that tolerant lines have developed responses to coping with the changes in membrane compositions to sustain photosynthesis under cold stress conditions.

Under the combination of AG and cold stress conditions, the common GO terms were nitrogen compound metabolic process, carbohydrate metabolic process, signaling process, microtubule-based process, and cytoskeletal process. The unique GO terms in the tolerant line included isoprenoid metabolic process, glucosyltransferase activity, and glutamine family amino acid metabolic process, while the unique GO terms in the susceptible line were GTP binding, GTPase activity and response to stress. Under the combination of AG and cold stress, the common GO terms with higher number of enriched genes in the tolerant line were oxidoreductase activity, peroxidase activity, microtubule motor activity, cellular ketone metabolic process, organic acid metabolic process, oxoacid metabolic process, carboxylic acid biosynthetic process, and lipid biosynthetic process. Other GO terms had more enriched genes in the susceptible line, including cytoskeleton, mitochondrial envelope, microtubule cytoskeleton, hydrolase activity, oxidoreductase activity, protein serine/threonine kinase activity, antioxidant activity, nitrogen compound metabolic process, protein metabolic process, regulation of the cellular metabolic process, regulation of the biosynthetic process, regulation of nitrogen compound metabolic process, regulation of nucleobase, nucleoside, nucleotide, and the nucleic acid metabolic process. The common GOs identified between AG, cold and combined stress included carbohydrate metabolic process, glucosyltransferase activity, regulation of nitrogen compound metabolic process, protein metabolic process, lipid metabolic process, cellular nitrogen compound biosynthetic process, lipid biosynthetic process, microtubule-based process, whereas isoprenoid biosynthetic process, isoprenoid metabolic process, cell redox homeostasis, and the glutamine family amino acid metabolic process were uniquely enriched only in combined stress of AG and cold conditions. Isoprenoids, naturally synthesized volatile compounds, were found to play significant roles in defense mechanisms against different abiotic stresses, including drought, heat, and cold [109, 110]. Isoprenoids alter the ROS response by direct reactions of isoprenoids with ROS, indirect modification of ROS, and stabilization of lipid membranes developing the capability of plants to cope with oxidative stress inside the cells. The induction of 35 genes related to metabolism, transport, signal transduction, and stress responses, including transcription factor genes such as *DREB1A*, *IRO2*, and *NAC5,* with external application of glutamine, was reported [111]. Glutamate was reported to amplify its signal and interact with other signaling pathways to regulate metabolism, growth, and defense responses in rice.

The comparison of enriched GO terms in the tolerant line across different stresses identified the enrichment of ATPase activity, NADP or NADPH binding, photosynthesis and thylakoid part exclusively under AG stress, enrichment of phospholipid metabolic process, starch synthase activity, mitochondrial lumen, regulation of signaling process and GTPase regulator activity exclusively under cold stress, and enrichment of isoprenoid metabolic process, protein-DNA complex, DNA repair, and cell redox homeostasis exclusively under the combination of AG and cold stress. Similarly, the comparison of enriched GO terms in the susceptible line depicted enrichment of photosynthesis, carboxylic acid transport, glucan metabolic process, glucosyltransferase activity, sulfur compound biosynthetic process exclusively under AG stress, enrichment of tetrapyrrole metabolic process, porphyrin metabolic process, hydrolase activity, and ATPase activity exclusively under cold stress, and enrichment of lipid binding, transferase activity, and cellular protein complex assembly under the combination of AG and cold stress.

### Genes and transcription factors

Under AG stress, the unique downregulated genes and transcription factors with the highest expression values were OS03G0309400 (pectin esterase) with -11.70 log twofold change (log2FC) value, followed by OS03G0427300 (glutelin), OS05G0305300 (pentatricopeptide), OS04G0121300 (OsSub35 - Putative Subtilisin homologue), OS10G0443800 (MYB family transcription factor), OS07G0147550 (photosystem II 10 kDa polypeptide, chloroplast precursor) ranging from -10.1 to -11.1 log2FC. Whereas the upregulated genes and transcription factors with the highest expression value (ranging from 11.6 to 30 log2FC) were OS10G0475000 (alcohol oxidase) with 30 log2FC value, followed by OS08G0463500 (C2H2 zinc finger protein), OS04G0606450 (ATP-dependent family protein), OS04G0103900 (chalcone synthase), OS06G0297400 (cadmium tolerance factor), OS10G0558600 (pentatricopeptide), OS08G0152400 (cytochrome P450), and OS10G0389300 (red chlorophyll catabolite reductase) (Supplementary Table 7).

Under cold stress, the unique downregulated genes and transcription factors with the highest expression (-7.1 to 12.3 log2FC) were OS10G0178000 (LTPL132, protease inhibitor/seed storage/LTP family protein precursor) with -12.3 log2FC followed by OS09G0457400 (alpha-amylase precursor), and OS04G0295400 (jasmonate-induced protein). The upregulated genes and transcription factors with the highest expression (6 to 11.1 log2FC) were OS07G0639400 (peroxidase precursor), OS07G0520300 (cytochrome P450 domain-containing protein), OS04G0604800 (glycosyl hydrolases family), and OS03G0155500 (expansin precursor).

In combination with AG and cold stress, the unique downregulated genes and transcription factors with the highest expression (-9.5 to -15.3 log2FC) were OS11G0635300 (cytochrome P450), OS09G0551400 (serine/threonine-protein kinase receptor precursor), and OS07G0525900 (chalcone and stilbene synthases). On the other hand, the unique upregulated genes 7.5 to 12.03 log2FC) were OS01G0512200 (AP-3 complex subunit delta), OS07G0431160 (F-box domain-containing protein), OS11G0484700 (Protease inhibitor/seed storage/LTP family protein precursor), OS11G0575600 (lipoxygenase), and OS06G0688200 (beta-expansin precursor).

Under AG stress, transcription factors (TFs) related to aldehyde dehydrogenase, cytochrome P450, dehydration responsive element binding protein (DREB), ethylene response factor (ERF), heat stress transcription factor, heat shock protein, alpha expansin, beta expansin, trehalose-6-phosphate synthase, trehalose-6-phosphate phosphatase, ATP binding cassette (ABC) transporters, AP2 domain-containing proteins, bHLH, bZIP, MYB, C2H2 zinc finger proteins, and WRKY were differentially expressed in both tolerant and susceptible lines (Supplementary Table 6). Under cold stress, the same transcription factors were expressed except for trehalose-6-phosphate phosphatase and beta expansin. The number of TFs from the DREB family and heat stress transcription family was higher. In contrast, TFs from cytochrome P450, aldehyde dehydrogenase, and trehalose-6-phosphate synthase were lower under cold stress than AG stress. Similarly, the same TFs were expressed under combined AG and cold stress while TFs from cytochrome P450, ethylene response factor (ERF), aldehyde dehydrogenase, alpha expansin, and beta expansin were expressed higher than under AG and cold stress. In addition, triose phosphate translocator 2 (OsTPT2) was expressed under AG stress and combined AG and cold stress. The TF, BBR-BPC, was uniquely expressed in both tolerant and susceptible lines under cold stress. The TF, YABBY, was expressed only in the tolerant line under AG stress and cold stress but not under combined AG and cold stress. We found aldehyde dehydrogenase, cytochrome P450, dehydration-responsive element binding protein (DREB), ethylene response factor (ERF), heat stress transcription factor, heat shock protein, alpha expansin, beta expansin, trehalose-6-phosphate synthase, trehalose-6-phosphate phosphatase, ABC transporters, AP2 domain-containing proteins, bHLH, bZIP, MYB, C2H2 zinc finger proteins, and WRKY expressed in both tolerant and susceptible line under AG stress, cold stress and combined AG and cold stress.

The ATP binding cassette (ABC) protein family consists of membrane proteins that are active in the ATP-powered transport of structurally unrelated substrates across the membrane [112–114]. The role of ABC transporters in plants in response to abiotic stress has not been widely explored. Previously, *Ospdr9*, a gene encoding an ABC protein, was reported to be induced by hypoxia stress in rice [115]. In recent years, ABC proteins have been identified as important elements in the transport of phytohormones, heavy metals, lipids, chlorophyll catabolites, and secondary metabolites [116]. ABC proteins in plastids play a key role in the biosynthesis of membrane lipids like galactolipids in the thylakoid membrane [116]. It has been previously reported that AP2/ERF genes, a large multigene superfamily of transcription factors, play an important role in waterlogging and flooding stress [117–119]. Hatorri et al. (2009) reported the deep-water response of two genes encoding ethylene response factors, *SNORKEL1* and *SNORKEL2,* in rice [118]. Under deep water conditions, ethylene accumulates in water and induces the expression of both genes, which ultimately lead to internode elongation via gibberellin. Hinz et al. (2010) reported that overexpression of *RAP2.2* gene belonging to AP2/ERF transcription factor improves plant survivability under hypoxia stress conditions [119]. MYB-related genes were found to be significantly upregulated in soybean seeding due to flooding stress [86].

In this study, we identified 13 genes of the MYB family upregulated in the tolerant line and downregulated in the susceptible line under AG stress (Supplementary Figure 2g). The MYB transcription factors contain the MYB domain that is highly conserved in all eukaryotes and involved in regulating a wide range of functions of MYB proteins [120]. Several studies have shown the involvement of MYB transcription factors in regulatory networks associated with plant growth and development [121–123]. Many other studies have suggested the significant role of MYB proteins in regulating plant responses to abiotic stress [124–127]. A study conducted in *O. coarcata* transcriptomic changes under salt and submergence stresses (alone or combined, compared to control conditions) found several transcription factors, including NAC, WRKY, and MYB gene family members upregulated in leaves, indicating extensive transcriptional regulation in stress responses [128]. MYB-related genes were found to be significantly upregulated in soybean seeding due to flooding stress [86]. A few genes belonging to the MYB family have been previously reported to be induced under cold stress [129, 130]. OsMYBS3 was identified to be repressing the DREB1/CBF-dependent cold signaling pathway in rice [130]. These genes might be involved in a new additional pathway that regulates cold adaptation in rice [131]. Our study identified several differentially regulated genes of the MYB family induced under cold stress in both genotypes. This suggests that MYB family members may play an important role in the regulatory pathway of cold tolerance in rice. The P2/EREB family contains a large number of plant-specific TFs and participates in abiotic stress responses in plants [132]. We also identified many genes in the AP2/EREB family differentially regulated under cold stress.

Many WRKY genes have been found to be transcriptionally regulated in response to abiotic stress treatments in rice [133, 134]. Three DEGs of WRKY genes (*Os03g0758900*, *Os11g0490900* and *Os01g0826400)* regulated by cold or H_2_O_2_ stress in a rice variety have been reported [61]. In our study, we found several genes of the WRKY family upregulated and downregulated in both genotypes under cold stress. Additionally, heat shock proteins have been reported to be involved in heat shock responsiveness in plants [135]. Our study identified a few genes related to heat shock factor and heat shock protein differentially regulated. But the role of heat shock-related proteins in the cold stress response of plants remains obscure.

In this study, we observed 11 genes of Cyst2/His2 (C2H2) upregulated in the tolerant line and downregulated in the susceptible line under AG stress (Supplementary Figure 2i). These findings suggest that some members of C2H2-type zinc finger proteins (ZFPs) are important regulators of ROS signaling in rice under abiotic stress. The C2H2-type ZFPs have 176 members in *Arabidopsis* and 1,889 members in rice, and it constitutes one of the largest families of transcriptional regulators in plants [136, 137]. C2H2-type ZFPs are important components in regulating plant growth, development, hormone responses, and tolerance to biotic and abiotic stresses [136, 138, 139]. In rice, several members of C2H2-type ZFPs, such as ZFP182, ZFP245, ZFP252, and ZFP179, are involved in the responses of rice to drought, salinity, and oxidative stresses [140–144]. Many genes regulated by different transcription factors were previously reported to be induced under cold stress. Kanneganti et al. (2008) reported the expression of OsiSAP8 (Os06g0612800) under cold stress [145]. Kong et al. 2006 have identified the expression of OsDOS (Os01g0192000) under drought stress [146]. In our study, we found Os01g0192000 belonging to zinc finger protein upregulated in both genotypes under cold stress with higher log2FC in the susceptible line (3.35) than in the tolerant line (1.89). This shows that this gene may be involved not only in drought stress but also in cold stress.

NAC TF genes have been previously reported to encode a set of plant-specific TFs involved in responses to biotic and abiotic stress [147–149]. The expression of NAC genes was found to be upregulated during cold stress [150], but in our study, we found both upregulated and downregulated NAC genes in both genotypes. This shows NAC genes have diverse roles in cold stress responses in plants. Many genes related to bHLH protein and bHLH transcription factors have also been discovered to be differentially regulated in our study. ICE1, a bHLH protein in *Arabidiopsis*, has been reported to specifically bind to the CBF3 promoter region and promote the expression of CBF3, which leads to improved tolerance to cold stress [151]. Differential regulation of Os030741100, a bHLH gene induced by cold stress [61] has also been reported. In this study, we identified more DEGs related to abiotic stress tolerance mechanisms in the stress-tolerant line than in the susceptible line. Functional analysis of the most promising candidate genes identified for stress tolerance in our study can be performed by both gain and loss of function analysis in future studies.

### GO analysis of DEGs under different stresses

In order to identify the GO terms enriched by DEGs of each treatment, GO analysis was carried out. Considering the FDR of 0.05 and five minimum number of mapping entries per GO, a total of 232, 306, and 300 GOs were enriched for all DEGs identified for AG stress, cold stress and combined AG and cold stress (Supplementary Table 8). A total of 106, 355 and 331 GOs were enriched for upregulated DEGs of AG stress, cold stress, and combined AG and cold stress. A total of 280, 218 and 242 GOs were enriched for downregulated DEGs of AG stress, cold stress, and combined AG and cold stress. Pairwise comparison of enriched GOs across different stress was performed to find common GOs. A total of 65 and 92 GOs were discovered to be shared between AG stress and cold stress from upregulated and downregulated genes, respectively. A total of 41 and 80 GOs were identified to be shared between AG stress and combined AG and cold stress from upregulated and downregulated DEGs, respectively. A total of 219 and 147 GOs were shared between cold stress and combined AG and cold stress from upregulated and downregulated DEGs, respectively. On the other hand, a total of 108, 37, and 108 GOs were uniquely enriched for upregulated genes in combined stress, AG stress, and cold stress, and a total of 78, 171 and 42 GOs were uniquely enriched for down-regulated genes, respectively. Some of these uniquely enriched GOs from upregulated DEGs of one of these stresses were found to be enriched by down-regulated DEGs of other stresses or vice versa.

**Fig. 2:**
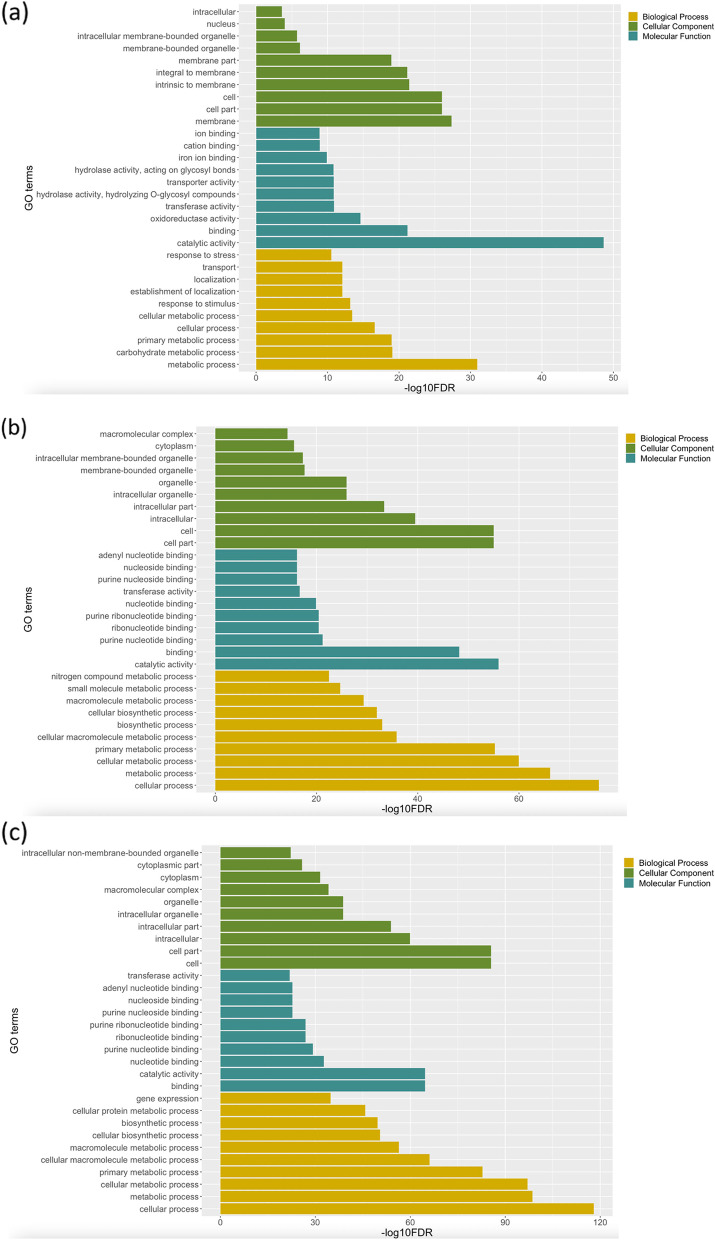
The top 10 gene ontology (GO) classification for DEGs across stresses for each category. GO terms from biological process, cellular component and molecular function categories enriched with DEGs under (**a**) anaerobic germination, (**b**) cold germination, and (**c**) combination of anaerobic and cold stress during germination conditions. The -log10 FDR indicates the enrichment score

The top 10 GO terms of each biological process, molecular function, and cellular component main categories with the highest enrichment scores for each treatment are shown in Fig. 2. Among the top 10 GOs, the common GOs between AG stress, cold stress and combined AG and cold stress, were cellular process, metabolic process, cellular metabolic process, primary metabolic process, catalytic activity, cell part, and cell membrane-bounded organelle and intracellular membrane-bounded organelle. Between AG stress and cold stress, three GO terms were found to be common among top ten GO. i.e., binding, membrane-bounded organelle, and intracellular membrane-bounded organelle. Among cold stress and combined AG and cold stress, 17 GO terms including cellular macromolecule metabolic process, biosynthetic process, cellular biosynthetic process, macromolecule metabolic process, purine nucleotide binding, purine ribonucleotide binding, ribonucleotide binding, transferase activity, purine nucleoside binding, nucleoside binding, adenyl nucleotide binding, intracellular, intracellular part, intracellular organelle, organelle, cytoplasm, and macromolecular complex were found to be common among top 30 GO belong to biological process, cellular component, and molecular function.

The pairwise comparison of enriched GOs of upregulated genes of cold stress and combined AG and cold stress showed enrichment of nitrogen compound metabolic process, carbohydrate metabolic process, response to stress, cellular protein metabolic process, protein metabolic process, cellular ketone metabolic process, cellular nitrogen compound metabolic process, cellular amino acid metabolic process, and lipid metabolic process. Among 147 common GO between cold stress and combined AG and cold stress from downregulated DEGs, post-translational protein modification, phosphate metabolic process, protein amino acid phosphorylation, protein serine/threonine kinase activity, phospholipid binding, and lipid binding were significantly enriched. The GO terms such as transcription, response to stress, nitrogen compound metabolic process, cellular biosynthetic process, RNA metabolic process, carbohydrate metabolic process, photosynthesis, ATP binding, carboxylic acid binding, and cell wall were commonly enriched among upregulated DEGs AG stress and cold stress. Similarly, the GO terms such as carbohydrate metabolic process, alcohol metabolic process, protein modification process, phosphorylation, cellular nitrogen compound metabolic process, lipid binding, lipid localization, lipid transport, cellular ketone metabolic process, glycogen metabolic process, tryptophan metabolic process were commonly enriched among downregulated DEGs AG stress and cold stress. Among AG stress and combined AG and cold stress, the commonly enriched GOs from upregulated genes included nitrogen compound metabolic process, carbohydrate metabolic process, purine nucleotide binding, purine ribonucleotide binding, and response to stimulus, and the commonly enriched GOs from downregulated genes included carbohydrate metabolic process, alcohol metabolic process, protein modification process, phosphate metabolic process, cellular nitrogen compound metabolic process, and lipid binding.

The common GO enriched by upregulated DEGs in AG condition and downregulated DEGs in cold condition included trehalose metabolic process, trehalose biosynthetic process, glycoside metabolic process, disaccharide biosynthetic process, while the GO enriched by downregulated DEGs in AG condition and upregulated DEGs in cold condition included carbohydrate catabolic process, alcohol catabolic process, glucose catabolic process, fructose-bisphosphate aldolase activity. The GO terms enriched by upregulated DEGs in cold conditions and downregulated DEGs in combined AG and cold stress were chromatin assembly or disassembly, nucleosome organization, chromosome organization, and DNA packaging. The GO terms enriched by upregulated DEGs in cold conditions and downregulated DEGs in AG conditions were glycolysis, carbohydrate catabolic process, alcohol catabolic process, phosphotransferase activity, and phosphate group as acceptor. The GO terms enriched by upregulated DEGs in the combined condition of AG and cold stress and downregulated DEGs in AG condition were polysaccharide biosynthetic process, fatty acid metabolic process, lipid biosynthetic process, and cellular lipid metabolic process. The GO terms enriched by upregulated DEGs in AG and downregulated DEGs in the combined condition of AG and cold stress were exocyst and cell cortex. The GO terms enriched by downregulated DEGs in AG and upregulated DEGs in the combined condition of AG and cold stress were cellular lipid metabolic process, fatty acid metabolic process, polysaccharide biosynthetic process, dicarboxylic acid metabolic process, and oxidoreductase activity.

Glycosyl hydrolases family 16, Os06g0697000, was upregulated in both AG stress and cold stress but downregulated under combined AG and cold stress. In contrast, the opposite trend was shown by another glycosyl hydrolases family 16, Os06g0696566. Glycoside hydrolases were identified to be involved in the hydrolysis of glycosidic bonds in complex sugars such as cellulose and starch [152, 153]. Glycosyl hydrolase 14 family gene, Os10g0565200, encoding beta-amylase, was reported to be significantly upregulated under cold stress [154]. Another glycosyl hydrolase gene, Os06g0675700, was significantly upregulated in the cold-tolerant line than in the cold-sensitive line [155]. Transcriptomics analysis of anther of contrasting genotypes revealed Os3BGlu7 [156] and GIF1 [157], encoding glycosyl hydrolase family 1 and glycosyl hydrolase family 32, respectively, were significantly upregulated in the cold susceptible genotype, LJ11 compared with cold-tolerant genotype, LJ25 under cold treatment [158]. The glycosidase hydrolase genes, Os11g0701400, Os11g0701600, Os11g0701900, and Os11g0702200 were significantly upregulated in the tolerant line under submergence [33]. Another member of the glycosyl hydrolase 14 family gene, Os10g0565200, was significantly upregulated under cold stress conditions [154], suggesting the potential involvement of the enzymes of the glycosyl hydrolase family on cold and AG tolerance.

Among the three common DEGs related to cellulose synthesis and representing cellulose synthase-like family A and family E, CSLA1(Os02g0192500) was upregulated under cold stress and combined AG and cold stress but downregulated under AG stress, whereas CSLE1 (Os09g0478100) and CSLE6 (Os09g0478300) were downregulated under all three stresses. High expression of cellulose synthase genes is reported to increase tolerance to biotic [95] and abiotic [96] stresses in plants. Endler et al. (2015) demonstrated that maintaining microtubule organization for cellulose synthase positioning and continuous cellulose deposition is critical for biomass production during salt stress in *Arabidosis* [97]. Sperotto et al. (2018) used cellulose staining and showed higher cellulose deposition in cell walls during cold treatment compared to normal temperatures [159]. This group also reported higher expression of cell-wall-related genes involved in cell-wall biosynthesis in the cold-tolerant line than in the cold-sensitive line, indicating the importance of cellulose synthase and cellulose-synthase like proteins on maintaining cellular integrity during cold stress. Kong et al. (2010) reported downregulation of proteins related to cell wall metabolism and structural modification, such as B-glucanase, B-glucosidase, B-galactosidase in wheat, suggesting the correlation of downregulated proteins with inhibition of cell wall elongation under flooding stress suppressing the growth of wheat [160]. Similar trends were observed in woody plants such as gray poplar, where the expression of genes encoding cellulose synthases was strongly down-regulated (up to 44-fold) in roots under flooded condition [87].

Two common DEGs across all stresses were upregulated in cold and combined AG and cold stress and were downregulated under AG stress: one related to phospholipid synthesis which is cyclopropane-fatty-acyl-phospholipid (Os06g0574100) and the other, glyceraldehyde-3-phosphate dehydrogenase (Os02g0171100), was involved in glycolysis. Previous studies have demonstrated the upregulation of phosphometabolic processes under cold stress in *Lotus japonicus*, and *Arabidopsis thaliana* [161–163]. Changes in membrane phospholipids were reported to help adapt to low-temperature environments [164].

### KEGG and MapMan analysis of DEGs

The KEGG pathway enrichment analysis was performed to determine the function of the differentially expressed genes (DEGs) [165, 166]. Among AG stress-responsive DEGs, 5571 DEGs were mapped to 102 biological pathways, 26 cellular components, and 71 molecular functions. Likewise, 7206 cold stress-responsive DEGs were mapped to 356 biological pathways, 89 cellular components, and 81 molecular functions, while 13279 combined stress-responsive DEGs were mapped to 500 biological pathways, 131 cellular components, and 112 molecular functions. The top 20 pathways enriched by AG, cold, and combined stress-responsive DEGs are shown in Table 2.

**Table 2:** Top 10 KEGG pathways with DEGs under each stress

**AG stress**		**Cold stress**		**Combined AG and Cold stress**	
**KEGG Pathway name**	**Enrichment FDR**	**KEGG Pathway name**	**Enrichment FDR**	**KEGG Pathway name**	**Enrichment FDR**
Hydrogen peroxide catabolic process	8.60E-06	Carboxylic acid metabolic process	3.10E-10	Amide biosynthetic process	4.30E-43
Detoxification	3.90E-07	Small molecule metabolic process	3.60E-17	Translation	7.10E-38
Anion transmembrane transport	1.50E-06	Oxoacid metabolic process	1.50E-10	Peptide biosynthetic process	7.20E-38
Response to toxic substance	3.10E-07	Organic acid metabolic process	1.80E-10	Peptide metabolic process	3.00E-34
Cellular response to toxic substance	7.60E-07	Carbohydrate metabolic process	3.10E-10	Cellular amide metabolic process	4.80E-38
Cellular detoxification	7.60E-07	Organonitrogen compound biosynthetic process	1.90E-13	Organonitrogen compound biosynthetic process	1.40E-51
Small molecule catabolic process	3.70E-06	Response to stress	6.10E-15	Small molecule metabolic process	7.10E-33
Cellular oxidant detoxification	1.40E-05	Response to chemical	3.80E-10	Cellular nitrogen compound biosynthetic process	3.70E-54
Response to oxidative stress	5.30E-06	Organic substance biosynthetic process	2.20E-26	Cellular biosynthetic process	1.10E-66
Response to chemical	1.90E-11	Cellular nitrogen compound biosynthetic process	1.90E-17	Organic substance biosynthetic process	8.20E-66
Carbohydrate metabolic process	4.40E-09	Response to stimulus	8.30E-18	Cellular macromolecule biosynthetic process	5.30E-45
Cellular response to chemical stimulus	1.40E-06	Cellular biosynthetic process	4.00E-25	Macromolecule biosynthetic process	9.00E-45
Transmembrane transport	4.70E-10	Biosynthetic process	4.00E-25	Biosynthetic process	2.10E-62
Oxidation-reduction process	1.90E-11	Cellular macromolecule biosynthetic process	2.10E-15	Cellular protein metabolic process	2.50E-53
Small molecule metabolic process	7.40E-08	Macromolecule biosynthetic process	1.30E-14	Gene expression	9.00E-42
Catabolic process	1.80E-07	Regulation of cellular process	2.90E-12	Cellular nitrogen compound metabolic process	1.20E-55
Response to stimulus	1.60E-09	Regulation of biological process	8.50E-12	Protein metabolic process	1.70E-48
Localization	1.80E-07	Biological regulation	6.00E-13	Response to stimulus	3.30E-31
Transport	6.70E-07	Cellular protein metabolic process	3.10E-10	Heterocycle metabolic process	5.70E-31
Establishment of localization	1.00E-06	Cellular nitrogen compound metabolic process	3.20E-12	Organic cyclic compound metabolic process	8.60E-30

**Fig. 3:**
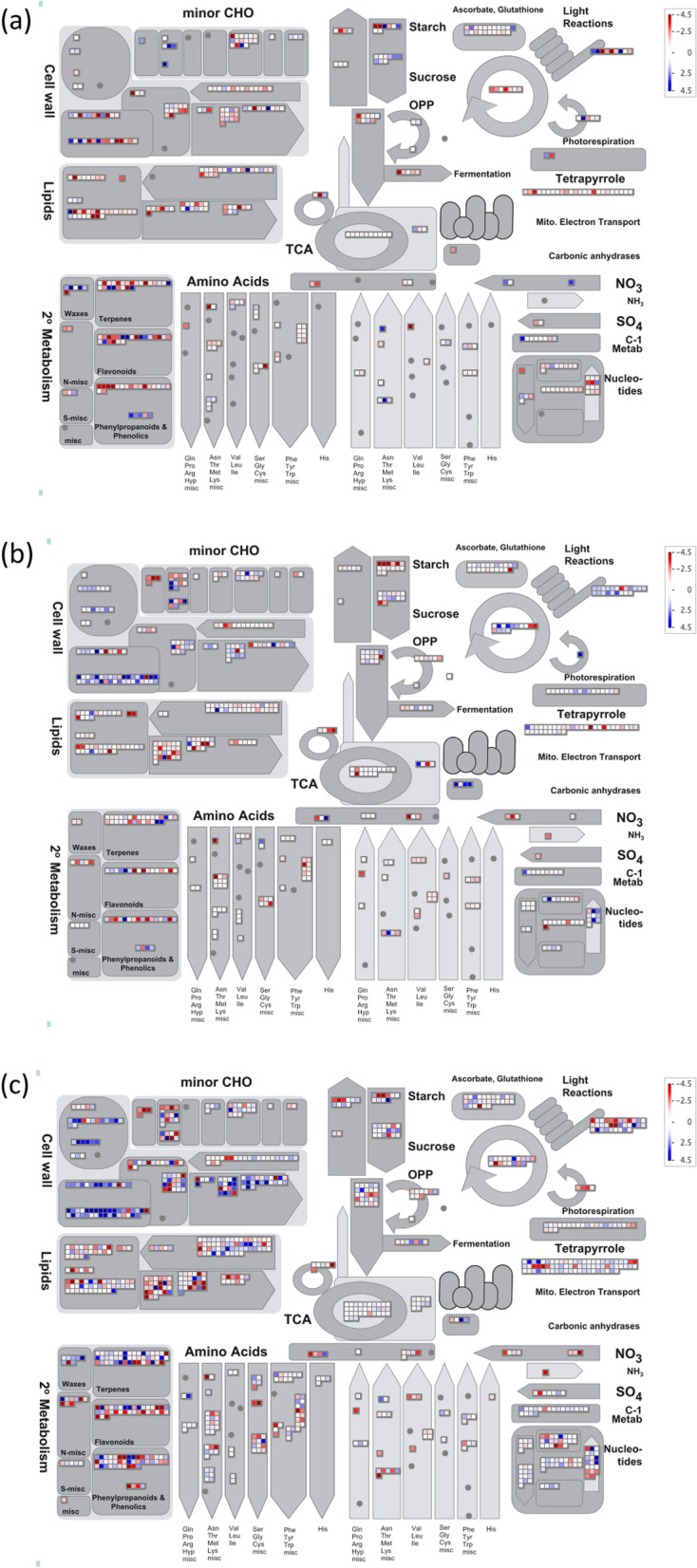
DEGs under different stress conditions were binned to MapMan metabolism bin. The MapMan diagrams for (**a**) anaerobic germination, (**b**) cold germination, and (**c**) combination of anaerobic and cold stress during germination are shown. Up-regulated and down-regulated transcripts are shown in blue and red, respectively

**Fig. 4:**
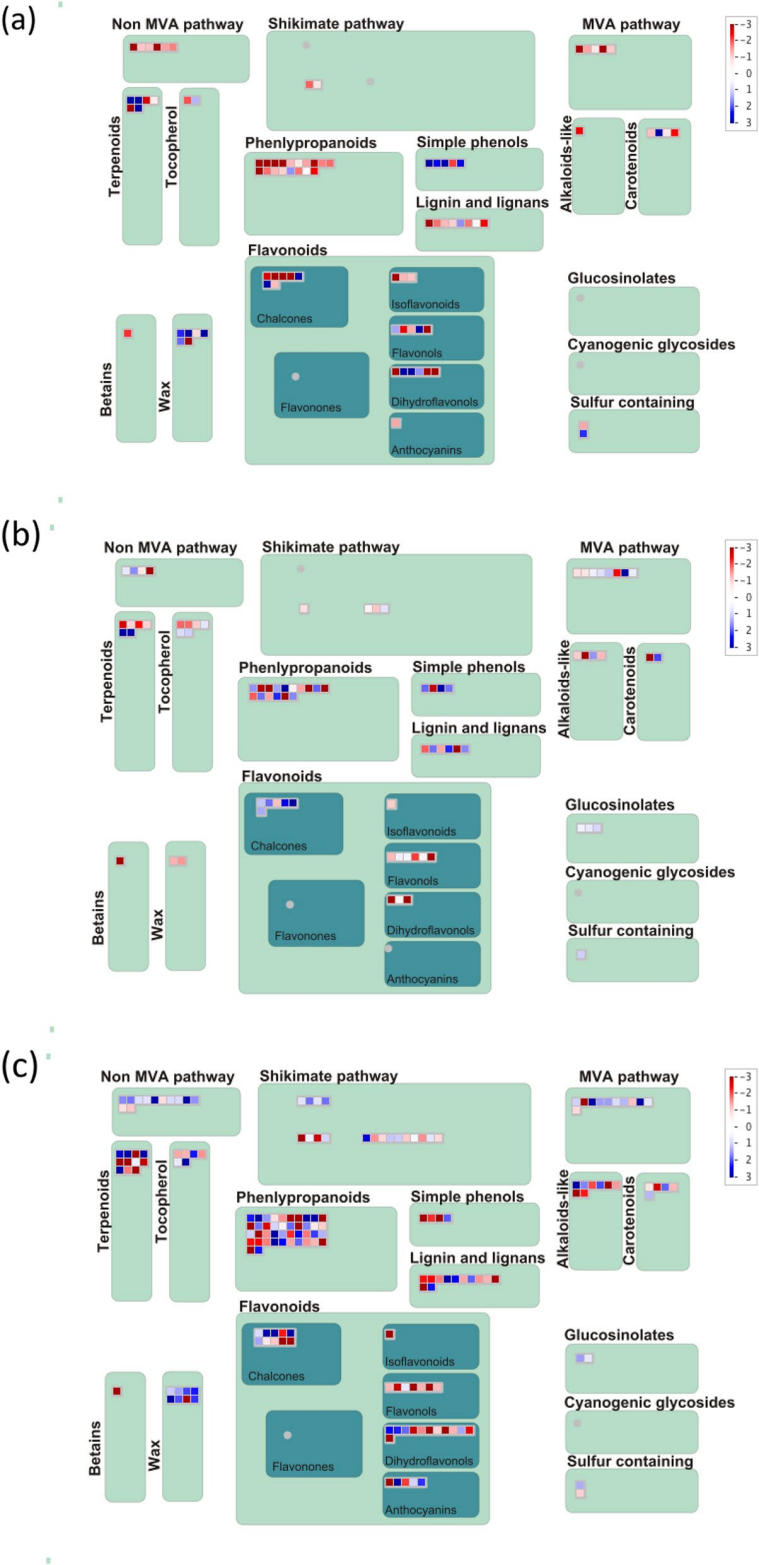
DEGs associated with secondary metabolism under different stress conditions were binned to MapMan functional categories. The MapMan diagrams for (**a**) anaerobic germination, (**b**) cold germination, and (**c**) combination of anaerobic and cold stress during germination conditions are shown. Up-regulated and down-regulated transcripts are shown in blue and red, respectively

As a complementary approach to the GO and KEGG analysis, MapMan analysis was also employed to categorize DEGs in bins and molecular pathways. Overall, AG stress-responsive DEGs were mapped to 84 bins (Fig. 3a). Secondary metabolism, minor aldehyde metabolism (trehalose), lipid metabolism, fermentation, and amino acid metabolism were highly enriched by DEGs under AG. Cold stress-responsive DEGs were mapped to 98 bins (Fig. 3b). Cell wall modification, light reaction, minor aldehyde metabolism (raffinose family), minor aldehyde metabolism (starch degradation family), and nucleotide metabolism were highly enriched by DEGs under cold stress. DEGs from combined AG and cold stress were mapped to 102 bins (Fig. 3c). Cell wall modification, mitochondrial electron transport (ATP synthesis), minor aldehyde metabolism (raffinose family), and lipid metabolism were highly enriched by DEGs under combined stress conditions. Cell wall degradation, cell wall modification, and minor aldehyde metabolism (raffinose family) were commonly enriched from DEGs from cold stress and combined AG and cold stress. Minor CHO metabolism (trehalose) and Calvin cycle were commonly enriched from DEGs under AG and cold stress. Amino acid metabolism and secondary metabolism were commonly enriched from DEGs from AG stress and combined AG and cold stress. The molecular pathways related to amino acid metabolism (synthesis of tryptophan, methionine), cell wall (cellulose synthesis), cell wall modification, fermentation, glycolysis, lipid metabolism (steroids, squalene, glycolipid synthesis), minor CHO metabolism (raffinose family, trehalose), N-metabolism (ammonia metabolism, nitrate metabolism), light reaction, and photorespiration were commonly enriched from DEGs from all three stresses. The number of DEGs differed among the common pathways identified with MapMan across different stresses. Additionally, the DEGs upregulated in one stress were downregulated in another stress for the same pathway. Among the genes related to cell wall modification, six DEGs related to expansin precursor and glycosyl hydrolase family were common across all stresses. Expansin precursors, Os03g0102500, Os04g0228400, Os10g0555600, and Os10g0556100, were upregulated under cold stress and combined cold and AG stress. In contrast, they were downregulated under AG stress. Expansins facilitate cell wall loosening by disrupting the hydrogen bonds between cellulose and xyloglucan polymers [167]. In a previous study, expansins were up-regulated at one or more time points of cold treatment in the tolerant line, NC567 and downregulated in susceptible line, Taiyan8 [168]. The upregulation of expansins in the roots of the drought-tolerant line, Nootripathu compared to drought susceptible line, IR20, was also reported [169]. Significant downregulation of the B-expansin gene (Os03g0106500), which regulates cell wall dynamics and cell size in rice, was also observed [170]. Separate MapMan analysis of each genotype under all three different types of stresses showed large number of enriched genes related to phenylpropanoids, phenomics, terpenes and flavonoids in both Darij and 4610 under combined AG and cold stress followed by AG stress and cold stress (Supplementary Figures 3, 4). A large number of upregulated genes were enriched in both Darij and 4610 under combined AG and cold stress whereas a large number of downregulated genes were enriched in both Darij and 4610 under AG and cold stress. For the secondary metabolism category, the common pathways enriched in all three stresses were phenylpropanoids, simple phenols, mevalonate (MVA), lignin biosynthesis, non-mevalonate pathway, terpenoids, and carotenoids (Fig. 4). The glucosinolates category was uniquely enriched for DEGs under cold stress (Fig. 4b) and combined AG and cold stress (Fig. 4c). Anthocyanins and tocopherol biosynthesis were enriched for AG stress (Fig. 4a) and combined AG and cold stress. Tyrosine was uniquely enriched for combined AG and cold stress. Separate MapMan analysis of secondary metabolism of Darij and 4610 under three different stress conditions showed large number of enriched genes related to lignins and lignans, and MVA pathway in combined stress followed by AG stress and cold stress (Supplementary Figs. 5, 6).

### Protein-protein interactions network of DEGs

**Fig. 5:**
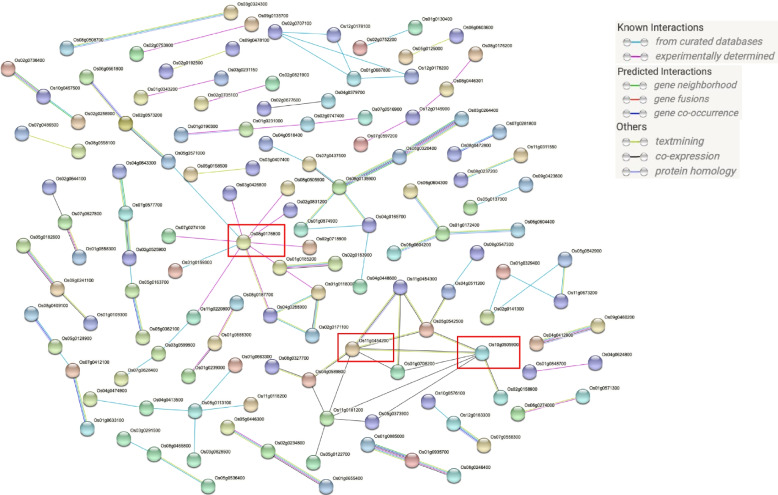
Hub genes identified by protein-protein interactions network. The hub genes are represented inside the red rectangular box

**Fig. 6:**
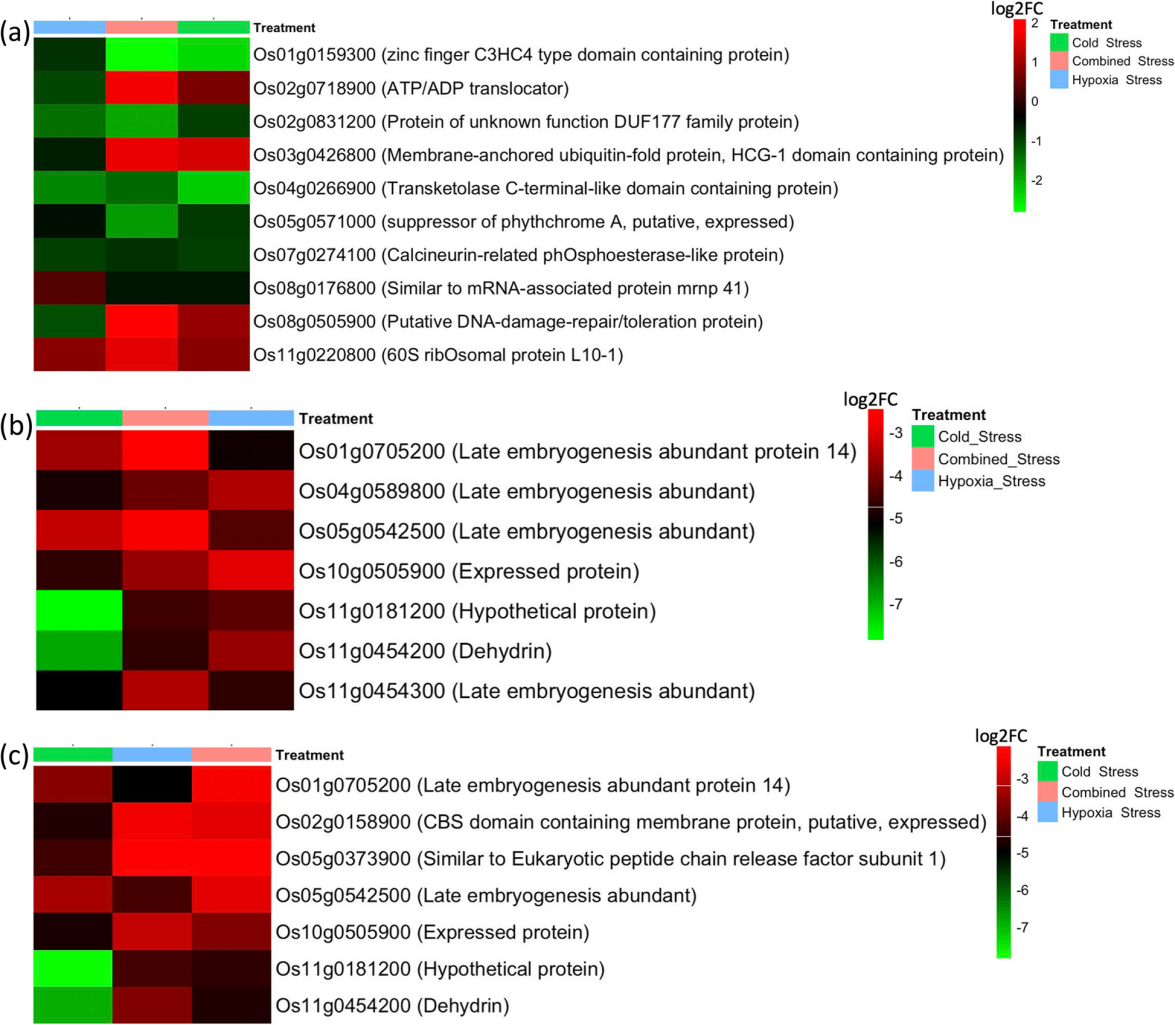
Heatmap showing log2FC of genes connected to the three hub genes. There were (**a**) ten genes connected to Os08g0176800, (**b**) seven genes connected to Os11g0454200, and (**c**) seven genes connected to OS10g0505900. Log2FC was estimated using DeSEQ2

A protein-protein interaction (PPI) network demonstrates various complex biological systems into a set of nodes and edges connecting the nodes. A total of 992 common DEGs identified among AG stress, cold stress and combined AG and cold stress were imported for protein-protein interactions. A high confidence score of 0.7 was used to construct the PPI. A hub gene was determined if the gene was connected to more than five genes. Three hub genes were identified in the data set (Fig. 5). Hub gene Os08g0176800 (similar to mRNA-associated protein mrnp 41), was found to be connected to 10 genes, including Os01g0159300 (zinc finger C3HC4 type domain containing protein), Os07g0274100 (calcineurin-related phosphoesterase-like protein), Os08g0505900 (putative DNA-damage-repair/toleration protein), and Os01g0159300 (zinc finger C3HC4 type domain-containing protein) (Fig. 6a). Another hub gene, Os11g0454200 (dehydrin) was found to be connected to seven other genes, including Os01g0705200 (late embryogenesis abundant protein 14), Os04g0589800 (late embryogenesis abundant, LEA), Os05g0542500 (LEA), and Os11g0454300 (LEA) (Fig. 6b). The last hub gene, Os10g0505900 (expressed protein) was found to be connected to seven genes, including Os01g0705200 (LEA protein 14), Os02g0158900 (CBS domain containing membrane protein, putative, expressed), Os05g0542500 (LEA), and Os11g0454200 (dehydrin) (Fig. 6c). The LEA protein family are unstructured polypeptides without a well-defined three-dimensional structure and have been reported to be significantly induced by abiotic stresses such as cold, drought, and salinity [171, 172]. The LEA protein family has been reported to be enhanced in drought tolerance by stabilizing cell membranes and protecting proteins [173]. The LEA protein family represents stress-induced hyper-hydrophilic proteins that accumulate in response to cellular dehydration. Previous research has postulated a positive correlation between the expression of LEAs and abiotic stress tolerance in plants [174, 175]. In rice, LEA genes of LEA_2, LEA_3, and dehydrin exhibited a strong response to osmotic stress (PEG), salt, and ABA [176]. OLE18, from the oleosin family, may have a structural role in stabilizing the lipid body during desiccation of the seed by preventing coalescence of the oil or providing recognition signals for specific lipase anchorage in lipolysis during seedling growth [177]. RAB16B and RAB21, from the plant dehydrin family, were known to be water stress-inducible proteins [178, 179].

## Conclusions

This study presents the findings of whole genome transcriptome profiles of two germinating rice genotypes that exhibit a contrasting response to flooding stress, cold stress, and combined flooding and cold stress. Our study highlights that the genes involved in carbohydrate metabolic process, glucosyltransferase activity, regulation of nitrogen compound metabolic process, protein metabolic process, lipid metabolic process, cellular nitrogen compound biosynthetic process, lipid biosynthetic process, and microtubule-based process were commonly enriched across all the stresses. Notably, we found that a significant number of DEGs have contributed to phenotypic differences in different stress conditions between the tolerant and susceptible genotypes. This study has provided the basis for identifying potential candidate genes involved in flooding, cold, and combined stress tolerance in germinating rice seed and possible use of the information for crop improvement.

## Materials and methods

### Plant materials and stress treatment

Two rice genotypes selected out of 257 accessions used for GWAS analysis and showing contrasting responses to anaerobic germination (AG) stress and cold stress [53] were selected. Darij was found to be tolerant, and 4610 was found to be susceptible, to both AG stress and cold stress during germination. All the seeds used in this study were freshly harvested and air-dried at 37°C for 5 days and then stored at 4°C prior to the experiment. In this experiment, we used seeds collected from single plants of each accession to maintain uniformity. Considering seed age effects on germination and vigor, we only used seeds that were about nine months old. The dormancy of seeds was broken by oven drying the seeds at 50°C for 5 days. The seeds were dehulled and surface sterilized with 70% ethanol for 5 mins, followed by rinsing with autoclaved distilled water three times. A total of ten replicates with 30 seeds/rep were used for each control and treatment in a completely randomized design for the phenotyping. The sterilized seeds were then germinated for 4 days on moist filter paper placed inside Petri dishes for phenotyping under control conditions. For submergence treatments, sterilized seeds were germinated in 8 cm deep water in glass beakers for 4 days.

For phenotyping under cold stress treatment, seeds were germinated in moist filter paper placed inside Petri dishes for 28 days in a refrigerator maintained at 13°C. A set of phenotypic traits, including shoot length under normal growth conditions (30°C) after 7 days after seeding (DAS), shoot length after 28 days of cold exposure (13°C), plumule recovery rate after 4 days of bringing the stressed seedlings back to normal temperature (30°C) was measured. Only seeds having coleoptile length half of the seed size were considered germinated [66]. For combined AG and cold stress treatment, sterilized seeds were germinated in 8 cm deep water in glass beakers at 13°C temperature for 28 and 35 days.

### Extraction of RNA and library construction

A total of four replicates with 30 seeds/rep were used for each control and treatment for the RNAseq samples. For preparing the samples for RNA extraction under cold stress, the surface-sterilized seeds were germinated in a growth chamber maintained at 13°C for 7 days in dark conditions. For the control samples, both the contrasting lines were germinated for 2 days in a growth chamber maintained at 30°C in dark conditions. By doing so, it is expected that the treated samples and the controls will have a similar physiological state since the growth of coleoptiles under cold stress are generally very slow. The germinating seeds with coleoptile length half the size of the seed were considered germinated. Germinating embryos from four biological replicates of each line grown under cold stress (7 DAS) and control conditions (2 DAS) were collected and immediately frozen in liquid nitrogen. Samples were then maintained at -80°C until RNA extraction. For samples under AG conditions, sterilized seeds of two contrasting lines were germinated in 8 cm deep water in a glass beaker for 4 days. For the control condition, sterilized seeds were germinated for 4 days on moist filter paper placed inside Petri dishes. Coleoptiles from four biological replicates of each line grown under AG stress (4 DAS) and normal condition (4 DAS) were collected and immediately frozen in liquid nitrogen and then maintained at -80°C until RNA extraction. For preparing samples for the RNA extraction under combination of AG stress and cold stress, sterilized seeds were germinated in 8 cm deep water in glass beakers at 13°C temperature for 7 days. For the control samples, both the contrasting lines were germinated for 2 days in a growth chamber maintained at 30°C in dark conditions. Germinating embryos from four biological replicates of each line grown under a combination of AG and cold stress (7 DAS) and under control (2 DAS) were collected and immediately frozen in liquid nitrogen, and the samples were then preserved at -80°C until RNA extraction. Any remaining embryos were included in the samples.

Total RNA was extracted using TRIzol reagent (Invitrogen, MA, USA) and QIAgen RNeasy Plant Mini Kit (Qiagen). RNA quality and concentration were checked using spectrophotometer NanoDrop ND-1000, and the integrity was checked using 1% agarose gel electrophoresis. Poly-A RNA containing mRNA was purified using poly-T oligo-attached magnetic beads and fragmented, and complementary DNA (cDNA) was synthesized using random hexamer primers, followed by purification, end-repairing, poly-A tailing, and adapter ligation. TruSeq Stranded RNA-seq libraries were prepared at Texas A&M AgriLife Genomics and Bioinformatics Service (TxGen; College Station, TX, USA) as per Standard Operating Protocol (Illumina). The quality of RNA libraries was further evaluated using Agilent 2100 Bioanalyzer (Santa Clara, CA, USA) using the kit Agilent DNA 1000. The libraries were run on multiple lanes of a HiSeq 4000 to generate 75 nt pair-end reads.

### RNA-seq analysis and differential expressed genes (DEGs)

FastQC was used for the analysis of the read quality and its visualization [180]. The low-quality bases and library adapters were removed from each library using Trimmomatic version 0.36 [181]. Reads were mapped against the reference genome of *Oryza sativa* cv. Nipponbare (IRGSP build 1.0 RAP-DB) [182, 183]. The mapping and alignment of the reads on the rice genome were done using HISAT2 version 2.1.0 [63]. Expression levels of each gene were quantified by normalizing total gene counts with the effective library size. DESeq2 v1.26 was used to test the pairwise differential expression analysis [184]. Genes with a *p*-value less than 0.05 were considered to be differentially expressed. We used the multi-factorial linear modeling and tested two null hypotheses of effects on gene expression: (1) whether stress treatment has a significant effect on the expression of each gene, and (2) whether gene expression was affected by stress treatment in a genotype-dependent manner. We fitted for models with our experimental data: (1) FM_trt_: *Y = *τ + ε; (2) FM_add_: *Y = *τ + γ + ε, and; (2) FM_full_: *Y* = τ + γ + τ:γ + ε. In each model, *Y* is the expression value of each gene, τ is the effect of stress treatment, γ is the effect of different genotype, and ε is the random error. Comparing FM_trt_ to FM_add_ separately, we tested whether the expression of each gene was regulated by stress, and whether there was a significant genotypic effect under stress. Comparisons of FM_full_ and FM_add_ allowed us to test whether gene expression was affected by stress in a genotype-dependent manner. In all cases, expression values for genes were standardized using the expression z=(x−x̅)/stdev for cross-genotype comparisons.

### Gene ontology and enrichment analysis

We used the web-based tool AGRIGO v2.0 to perform Gene Ontology (GO) enrichment analysis on DEGs [185], while the Fisher’s exact test and Benjamini-Hochberg’s false discovery rate (FDR) adjustment were used to control for multiple comparisons [186]. The GO term with FDR < 0.05 was regarded as significantly enriched. KEGG (Kyoto Encyclopedia of Genes and Genomes) pathway analysis was performed using ShinyGO v0.75 [187]. MapMan 3.5.1 software was applied to map DEGs to *O. sativa* pathways to determine genes involved in specific pathways [188].

### Protein-protein interactions network of DEGs

Common DEGs detected in AG stress, cold stress, and a combination of AG and cold stress were used to construct protein-protein interactions (PPIs) network. The STRING v11.5 database was used to detect both validated and predicted protein-protein interactions [189]. The resulting interactions were used to construct the PPIs network that was further analyzed and visualized using Cytoscape v3.7.0 [190]. PPIs network was constructed to identify hub genes (the nodes with the highest degree) involved in different stresses.

## Supplementary Information

**Additional file 1: Supplementary Figure 1.** PCA plot generated using regularized log transformation of total gene counts with DESeq2 under (a) flooding during germination, (b) cold stress, and (c) combined of flooding and cold stress during germination conditions. **Supplementary Figure 2.** Differentially expressed genes (DEGs) under flooding during germination conditions. (a) DEGs of ABC transporter family; (b) DEGs of AP2 domain containing protein family; (c) DEGs of helix-loop-helix DNA-binding domain containing protein family; (d) DEGs of pentatricopeptide (PPR) gene family; (e) DEGs of no apical meristem (NAM) protein family; (f) DEGs of RNA binding family; (g) DEGs of MYB transcription factor family; (h) DEGs of WRKY family; and (i) DEGs of C2H2 zinc finger protein family. **Supplementary Figure 3.** DEGs of Darij under a) flooding during germination, (b) cold stress, and (c) combined of flooding and cold stress during germination conditions were binned to MapMan metabolism bin. Up-regulated and down-regulated transcripts are shown in blue and red, respectively. **Supplementary Figure 4.** DEGs of 4610 under a) flooding during germination, (b) cold stress, and (c) combined of flooding and cold stress during germination conditions were binned to MapMan metabolism bin. Up-regulated and down-regulated transcripts are shown in blue and red, respectively. **Supplementary Figure 5.** DEGs of Darij under a) flooding during germination, (b) cold stress, and (c) combined of flooding and cold stress during germination conditions associated with secondary metabolism were binned to MapMan functional categories. Up-regulated and down-regulated transcripts are shown in blue and red, respectively. **Supplementary Figure 6.** DEGs of 4610 under a) flooding during germination, (b) cold stress, and (c) combined of flooding and cold stress during germination conditions associated with secondary metabolism were binned to MapMan functional categories. Up-regulated and down-regulated transcripts are shown in blue and red, respectively.

**Additional file 2: Supplementary Table 1.** Summary of RNA seq data and sequence assembly of control samples for flooding during germination stress. **Supplementary Table 2.** Summary of RNA seq data and sequence assembly of samples under flooding during germination stress. **Supplementary Table 3.** Summary of RNA seq data and sequence assembly of control samples for cold stress. **Supplementary Table 4.** Summary of RNA seq data and sequence assembly of samples under cold stress. **Supplementary Table 5.** Summary of RNA seq data and sequence assembly of samples under combined flooding and cold stress during germination conditions.

**Additional file 3: Supplementary Table 6.** Differentially expressed genes (DEGs) identified independently for each genotype (Darij (AG1) and line 4610 (AG2)) and each stress condition (germinating seeds under flooding (anaerobic germination, AG).

**Additional file 4: Supplementary Table 7.** Differentially expressed genes (DEGs) identified by considering both genotype and treatment effects.

**Additional file 5: Supplementary Table 8.** GO enrichment analysis in each stress condition.

## Data Availability

All the data supporting the results of this article are provided within the article or in the additional files.
