# Supplementary material for: Comparative transcriptomic analysis of germinating rice seedlings to individual and combined anaerobic and cold stress
Source: BMC Genomics. 2023 Apr 6;24:185. doi: 10.1186/s12864-023-09262-z (PMC10080786; doi:10.1186/s12864-023-09262-z)
Supplement: Supplementary file 1 — Additional file 1: Supplementary Figure 1. PCA plot generated using regularized log transformation of total gene counts with DESeq2 under (a) flooding during germination, (b) cold stress, and (c) combined of flooding and cold stress during germination conditions. Supplementary Figure 2. Differentially expressed genes (DEGs) under flooding during germination conditions. (a) DEGs of ABC transporter family; (b) DEGs of AP2 domain containing protein family; (c) DEGs of helix-loop-helix DNA-binding domain containing protein family; (d) DEGs of pentatricopeptide (PPR) gene family; (e) DEGs of no apical meristem (NAM) protein family; (f) DEGs of RNA binding family; (g) DEGs of MYB transcription factor family; (h) DEGs of WRKY family; and (i) DEGs of C2H2 zinc finger protein family. Supplementary Figure 3. DEGs of Darij under a) flooding during germination, (b) cold stress, and (c) combined of flooding and cold stress during germination conditions were binned to MapMan metabolism bin. Up-regulated and down-regulated transcripts are shown in blue and red, respectively. Supplementary Figure 4. DEGs of 4610 under a) flooding during germination, (b) cold stress, and (c) combined of flooding and cold stress during germination conditions were binned to MapMan metabolism bin. Up-regulated and down-regulated transcripts are shown in blue and red, respectively. Supplementary Figure 5. DEGs of Darij under a) flooding during germination, (b) cold stress, and (c) combined of flooding and cold stress during germination conditions associated with secondary metabolism were binned to MapMan functional categories. Up-regulated and down-regulated transcripts are shown in blue and red, respectively. Supplementary Figure 6. DEGs of 4610 under a) flooding during germination, (b) cold stress, and (c) combined of flooding and cold stress during germination conditions associated with secondary metabolism were binned to MapMan functional categories. Up-regulated and down-regulated transcripts are s [file 12864_2023_9262_MOESM1_ESM.pdf]

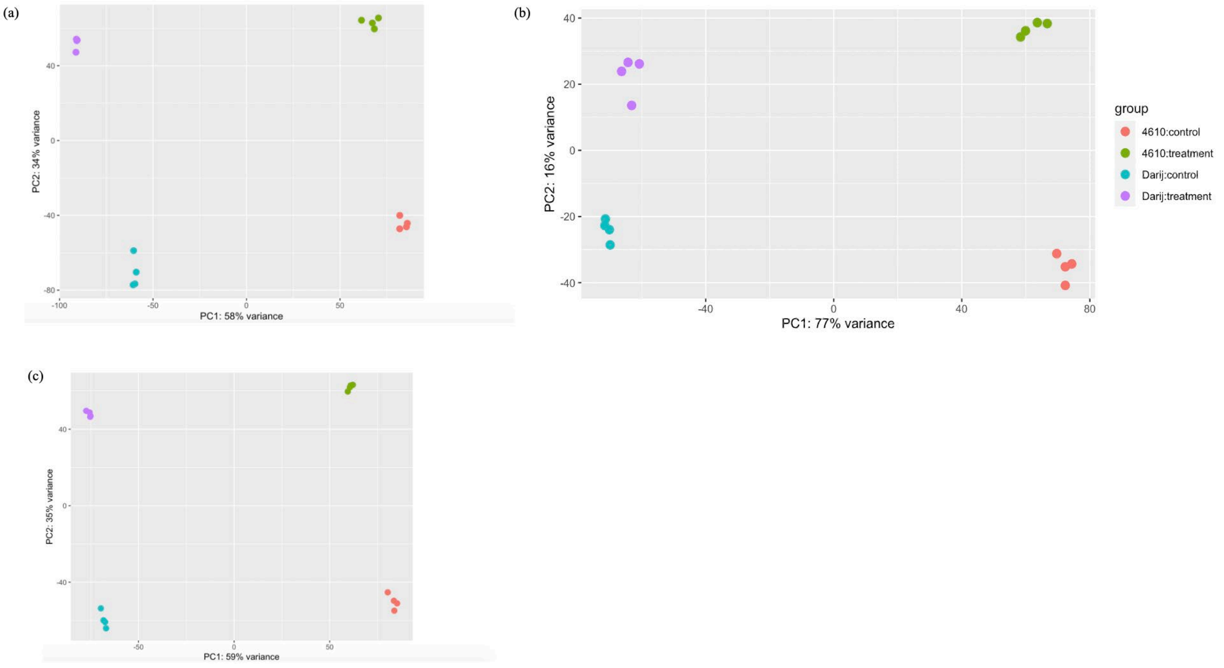


**Supplementary Figure 1. PCA plot generated using regularized log transformation of total gene counts with DESeq2 under (a) flooding during germination, (b) cold stress, and (c) combined of flooding and cold stress during germination conditions.**

a)

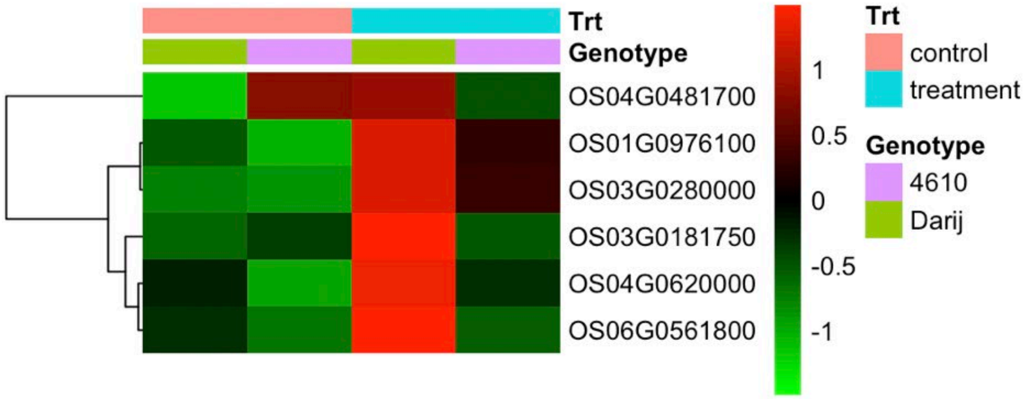
Trt
Genotype
OS04G0481700
OS01G0976100
OS03G0280000
OS03G0181750
OS04G0620000
OS06G0561800
1
0.5
0
-0.5
-1
Trt
control
treatment
Genotype
4610
Darij

(b)

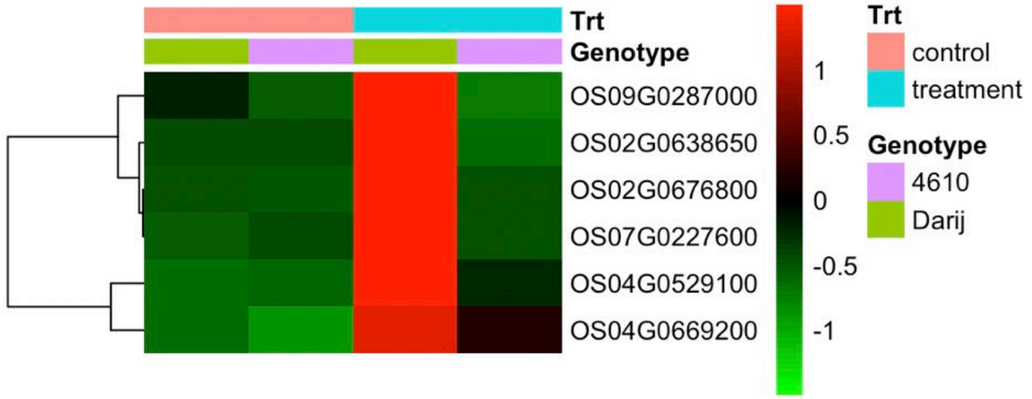
Trt
Genotype
OS09G0287000
OS02G0638650
OS02G0676800
OS07G0227600
OS04G0529100
OS04G0669200
1
0.5
0
-0.5
-1
Trt
control
treatment
Genotype
4610
Darij

(c)

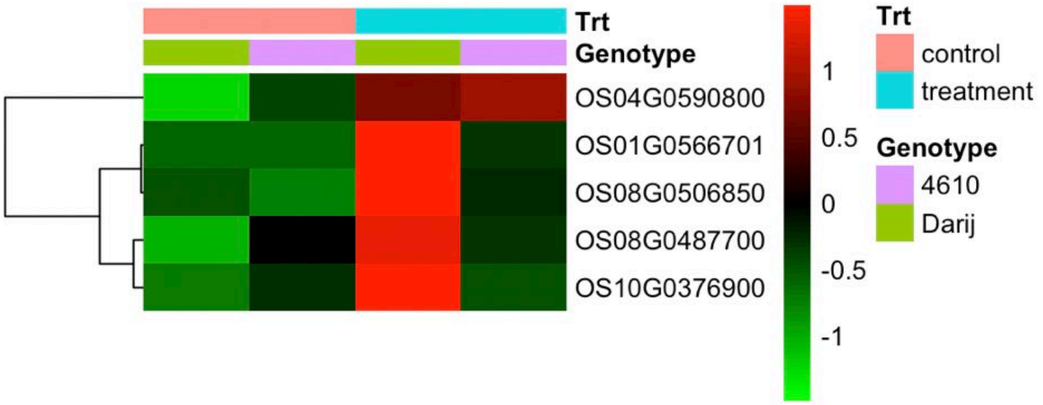
Trt
Genotype
OS04G0590800
OS01G0566701
OS08G0506850
OS08G0487700
OS10G0376900
1
0.5
0
-0.5
-1
Trt
control
treatment
Genotype
4610
Darij

**(d)**

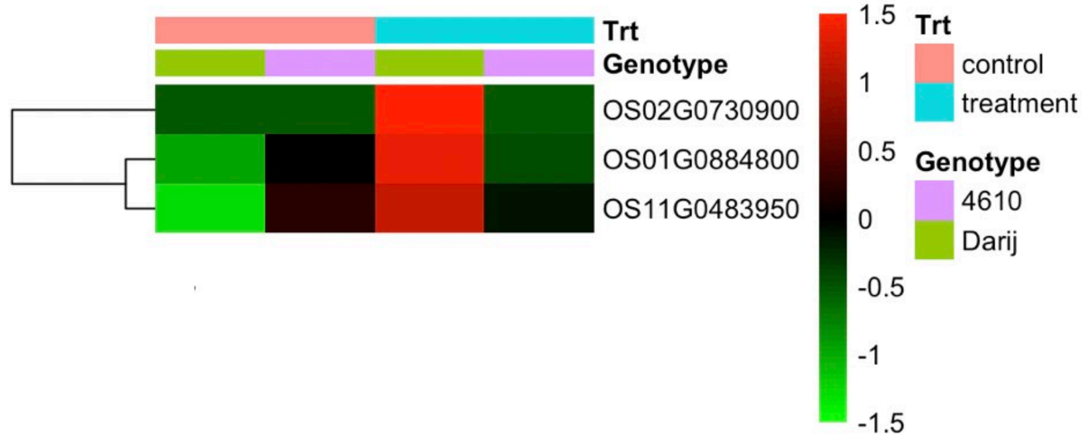
Trt
Genotype
OS02G0730900
OS01G0884800
OS11G0483950
1.5
1
0.5
0
-0.5
-1
-1.5
Trt
control
treatment
Genotype
4610
Darij

**(e)**

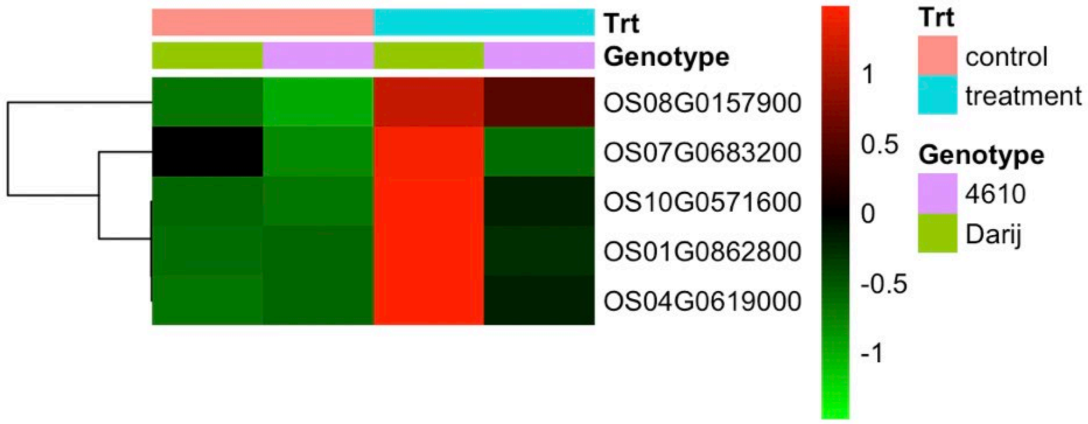
Trt
Genotype
OS08G0157900
OS07G0683200
OS10G0571600
OS01G0862800
OS04G0619000
1
0.5
0
-0.5
-1
Trt
control
treatment
Genotype
4610
Darij

**(f)**

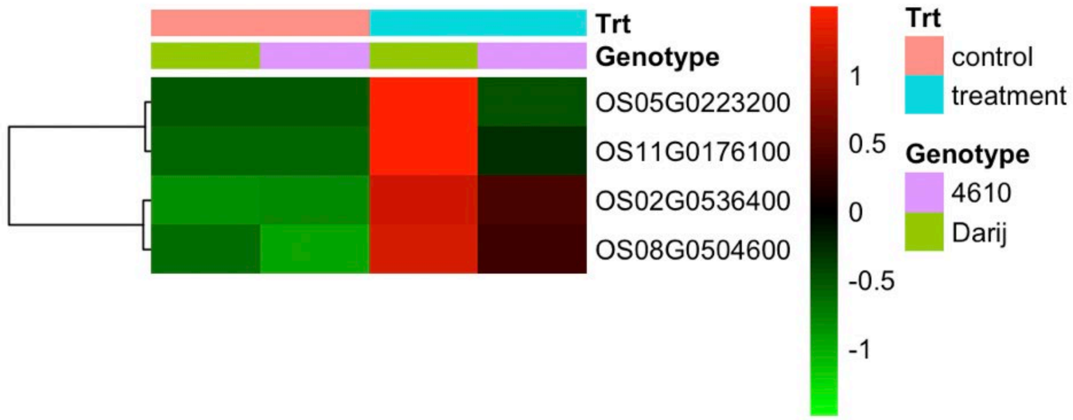
Trt
Genotype
OS05G0223200
OS11G0176100
OS02G0536400
OS08G0504600
1
0.5
0
-0.5
-1
Trt
control
treatment
Genotype
4610
Darij

(g)

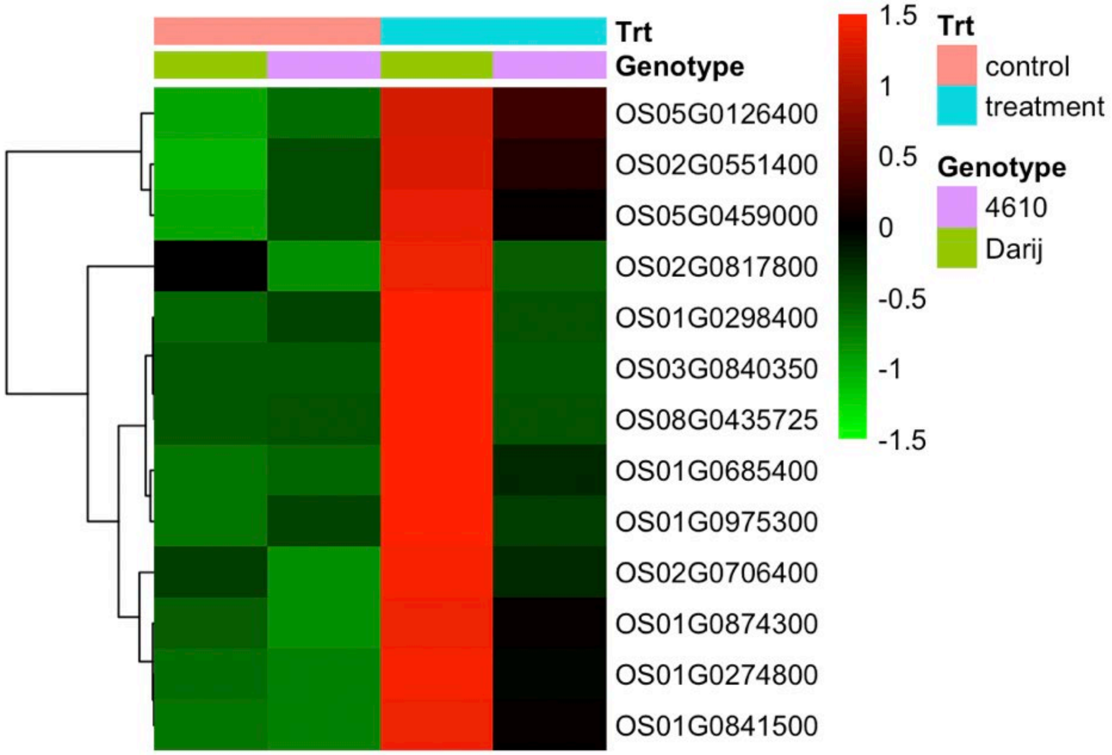
Trt
Genotype
OS05G0126400
OS02G0551400
OS05G0459000
OS02G0817800
OS01G0298400
OS03G0840350
OS08G0435725
OS01G0685400
OS01G0975300
OS02G0706400
OS01G0874300
OS01G0274800
OS01G0841500
1.5
1
0.5
0
-0.5
-1
-1.5
Trt
control
treatment
Genotype
4610
Darij

(h)

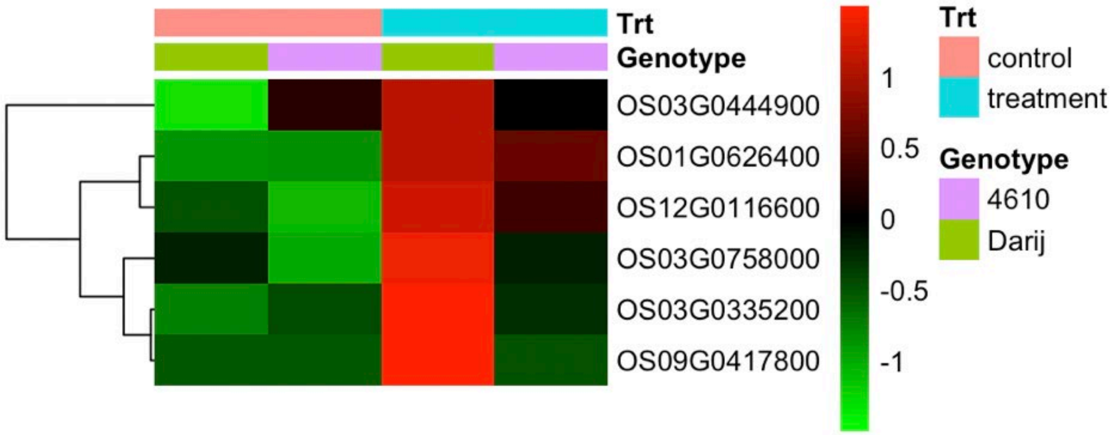
Trt
Genotype
OS03G0444900
OS01G0626400
OS12G0116600
OS03G0758000
OS03G0335200
OS09G0417800
1
0.5
0
-0.5
-1
Trt
control
treatment
Genotype
4610
Darij

**(i)**

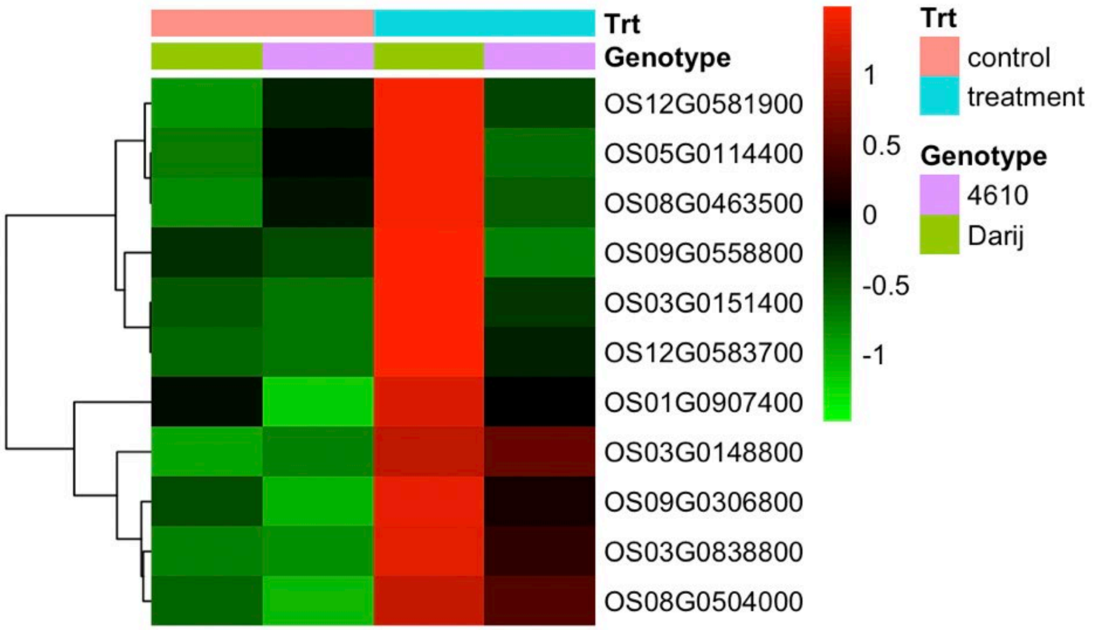


**Supplementary Figure 2. Differentially expressed genes (DEGs) under flooding during germination conditions.** (a) DEGs of ABC transporter family; (b) DEGs of AP2 domain containing protein family; (c) DEGs of helix-loop-helix DNA-binding domain containing protein family; (d) DEGs of pentatricopeptide (PPR) gene family; (e) DEGs of no apical meristem (NAM) protein family; (f) DEGs of RNA binding family; (g) DEGs of MYB transcription factor family; (h) DEGs of WRKY family; and (i) DEGs of C2H2 zinc finger protein family.

(a)

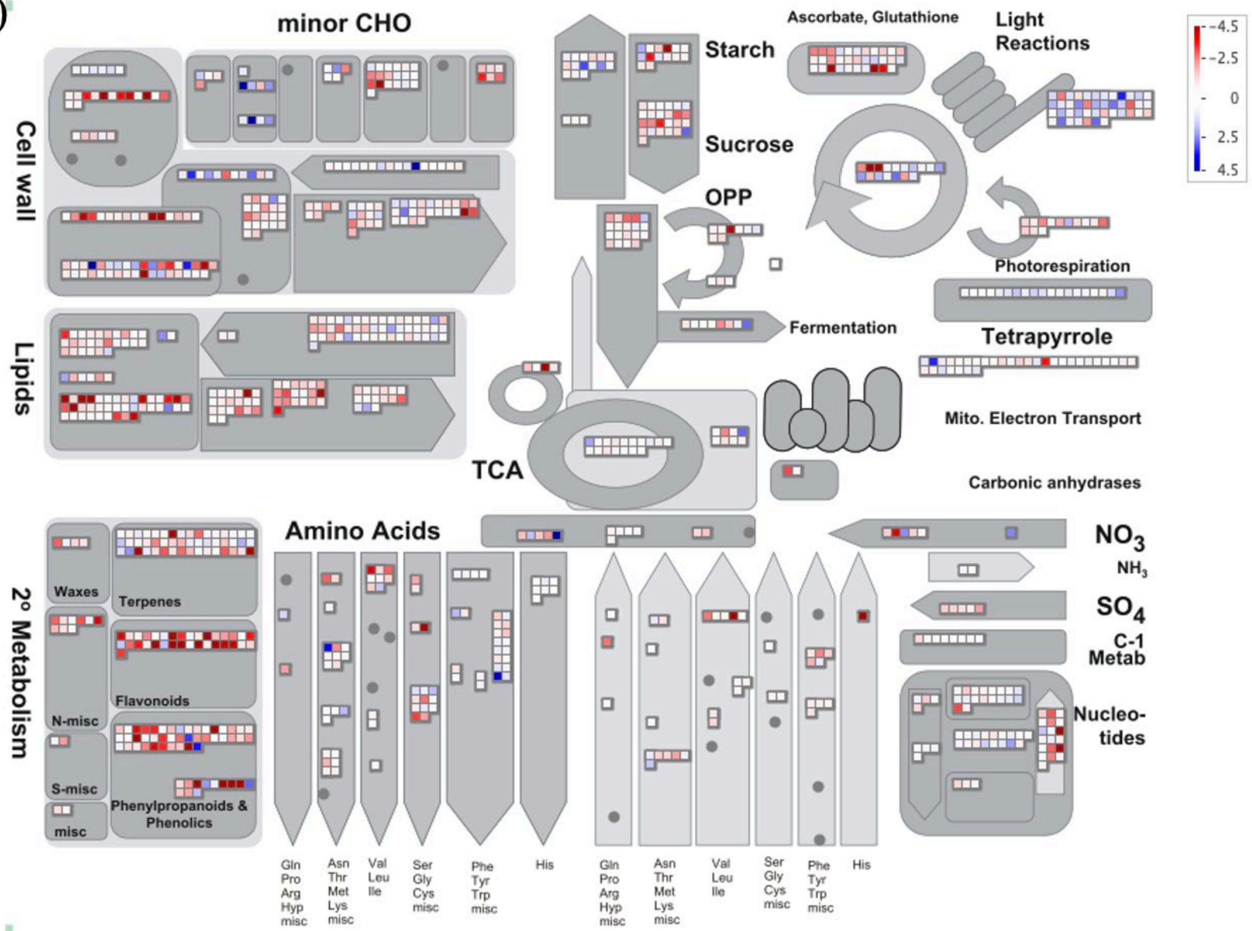
minor CHO
Cell wall
Starch
Sucrose
OPP
Ascorbate, Glutathione
Light Reactions
Photorespiration
Fermentation
Tetrapyrrole
Lipids
Mito. Electron Transport
TCA
Carbonic anhydrases
Amino Acids
NO3
NH3
SO4
C-1 Metab
Nucleo-tides
2° Metabolism
Waxes
Terpenes
N-misc
Flavonoids
S-misc
misc
Phenylpropanoids & Phenolics
Gln Pro Arg Hyp misc
Asn Thr Met Lys misc
Val Leu Ile
Ser Gly Cys misc
Phe Tyr Trp misc
His
-4.5
-2.5
0
2.5
4.5

(b)

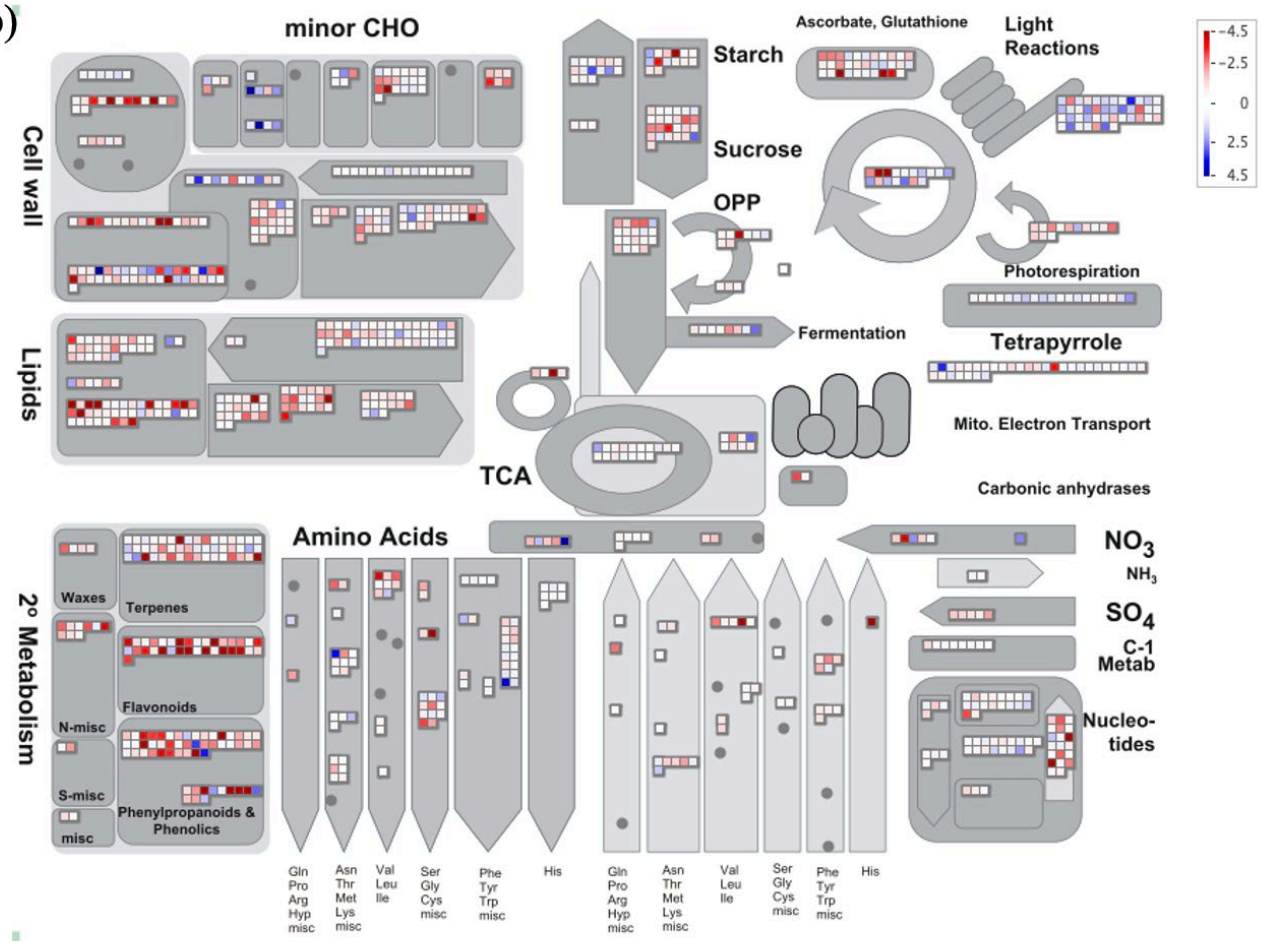
minor CHO
Cell wall
Lipids
Starch
Sucrose
OPP
Fermentation
TCA
Ascorbate, Glutathione
Light Reactions
Photorespiration
Tetrapyrrole
Mito. Electron Transport
Carbonic anhydrases
-4.5
-2.5
0
2.5
4.5
2° Metabolism
Waxes
Terpenes
N-misc
Flavonoids
S-misc
misc
Phenylpropanoids & Phenolics
Amino Acids
Gln Pro Arg Hyp misc
Asn Thr Met Lys misc
Val Leu Ile
Ser Gly Cys misc
Phe Tyr Trp misc
His
NO3
NH3
SO4
C-1 Metab
Nucleo-tides

(c)

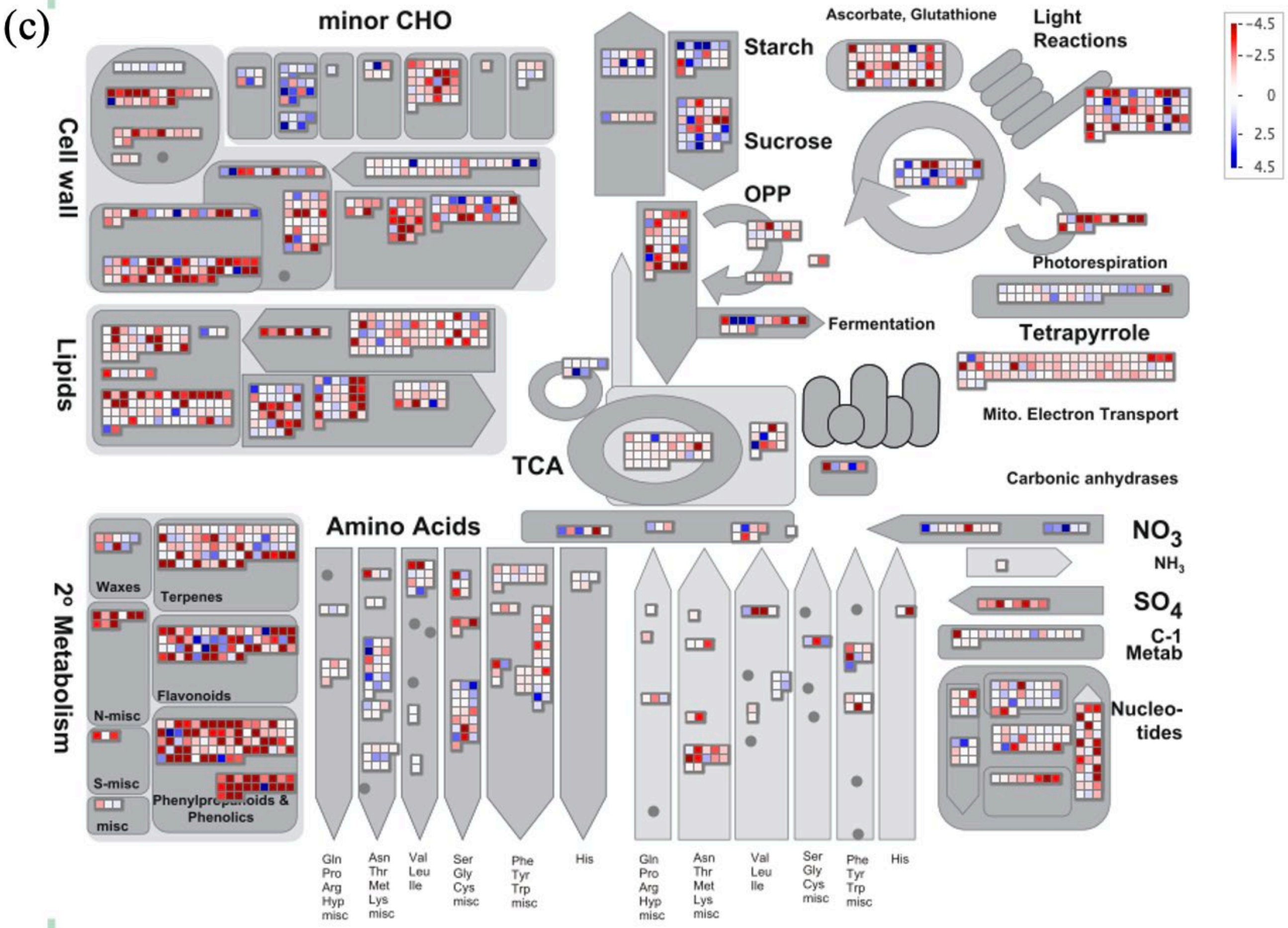


**Supplementary Figure 3. DEGs of Darij under a) flooding during germination, (b) cold stress, and (c) combined of flooding and cold stress during germination conditions were binned to MapMan metabolism bin.** Up-regulated and down-regulated transcripts are shown in blue and red, respectively.

(a)

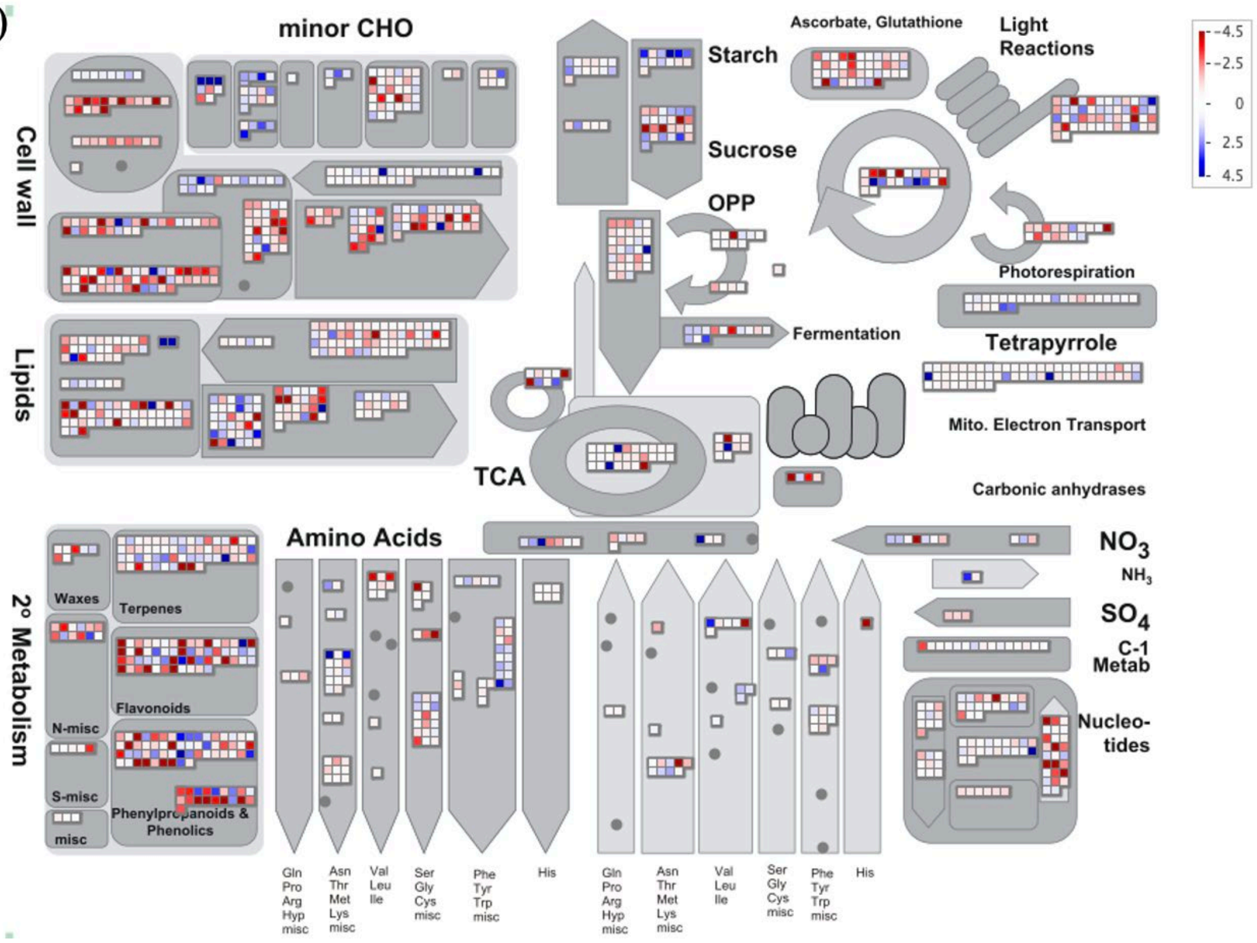

minor CHO
Cell wall
Lipids
2° Metabolism
Waxes
Terpenes
N-misc
Flavonoids
S-misc
Phenylpropanoids & Phenolics
misc
Amino Acids
Starch
Sucrose
OPP
Fermentation
TCA
Ascorbate, Glutathione
Light Reactions
Photorespiration
Tetrapyrrole
Mito. Electron Transport
Carbonic anhydrases
NO3
NH3
SO4
C-1 Metab
Nucleo- tides
-4.5
-2.5
0
2.5
4.5
Gln Pro Arg Hyp misc
Asn Thr Met Lys misc
Val Leu Ile
Ser Gly Cys misc
Phe Tyr Trp misc
His

(b)

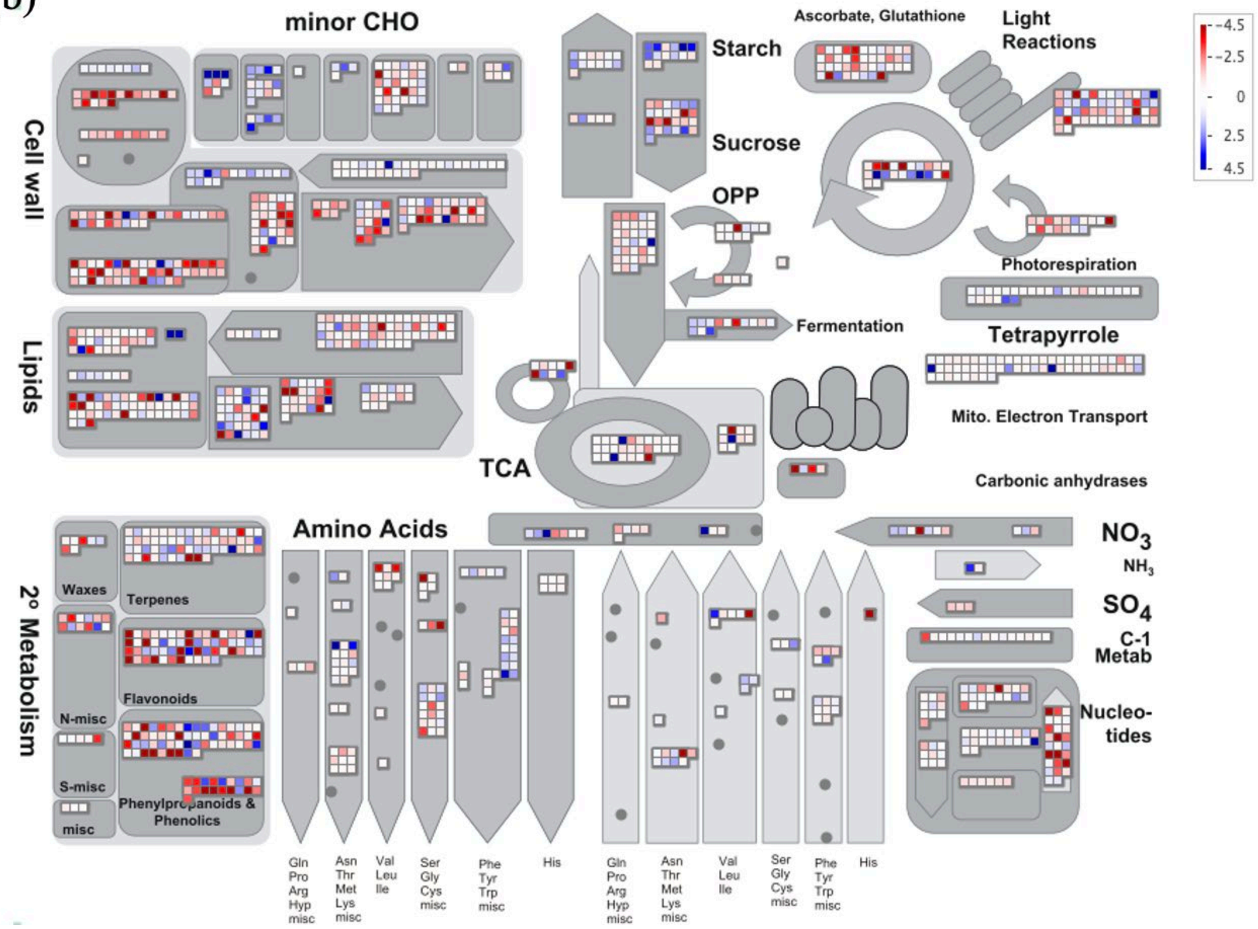

minor CHO
Cell wall
Lipids
Starch
Sucrose
OPP
Ascorbate, Glutathione
Light Reactions
Photorespiration
Fermentation
Tetrapyrrole
Mito. Electron Transport
Carbonic anhydrases
TCA
Amino Acids
2° Metabolism
Waxes
Terpenes
N-misc
Flavonoids
S-misc
misc
Phenylpropanoids & Phenolics
$NO_3$
$NH_3$
$SO_4$
C-1 Metab
Nucleo-tides
Gln Pro Arg Hyp misc
Asn Thr Met Lys misc
Val Leu Ile
Ser Gly Cys misc
Phe Tyr Trp misc
His
-4.5
-2.5
0
2.5
4.5

(c)

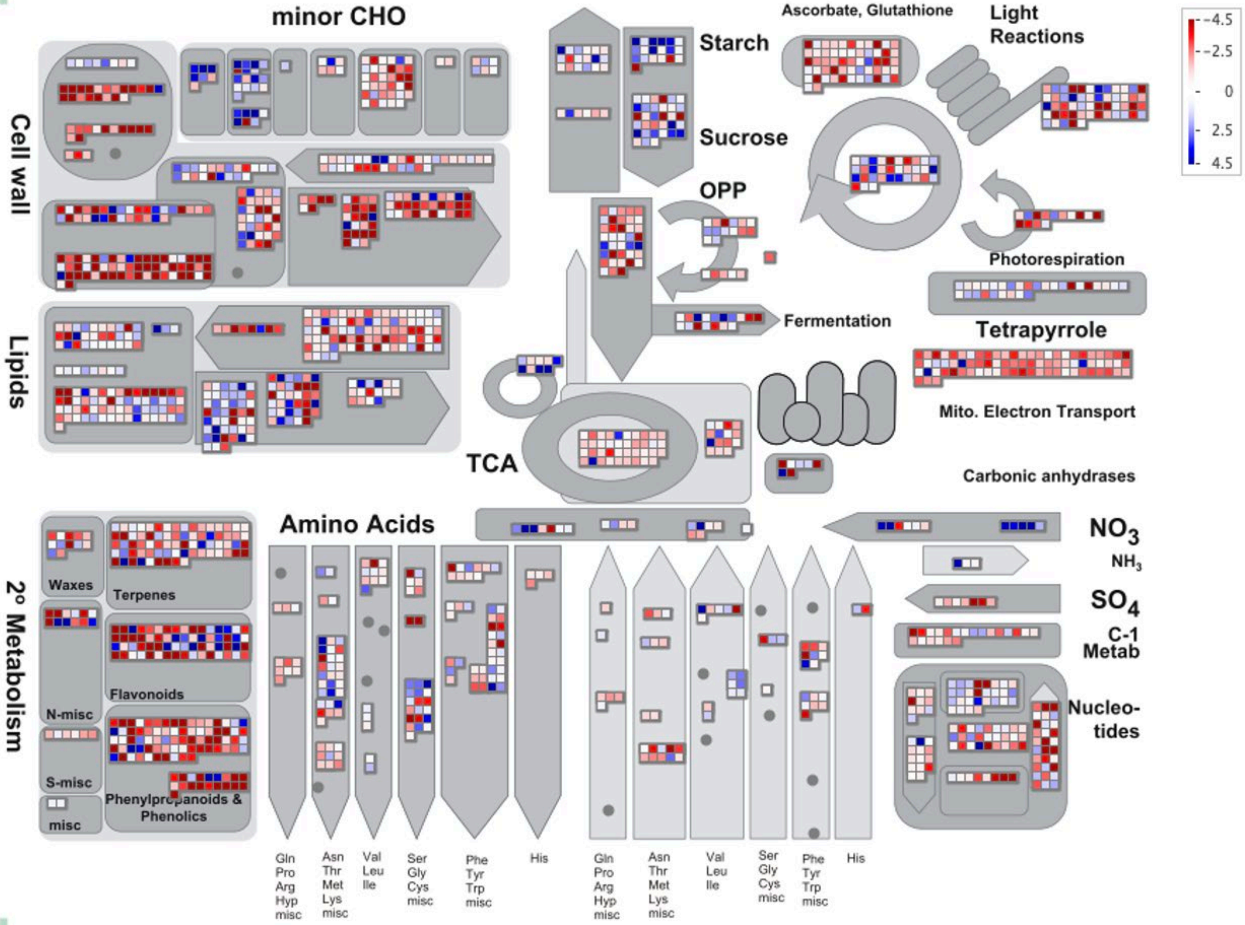


**Supplementary Figure 4. DEGs of 4610 under a) flooding during germination, (b) cold stress, and (c) combined of flooding and cold stress during germination conditions were binned to MapMan metabolism bin**. Up-regulated and down-regulated transcripts are shown in blue and red, respectively.

(a)

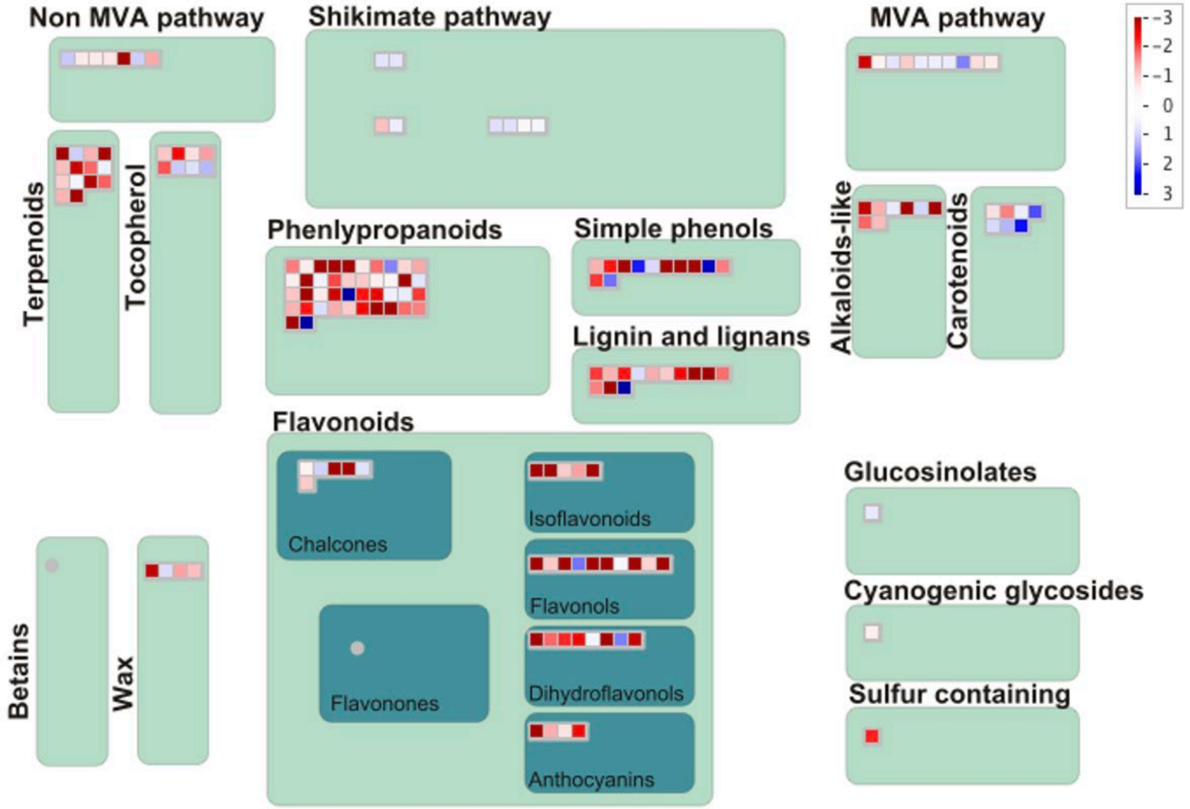

Non MVA pathway
Shikimate pathway
MVA pathway
-3
-2
-1
0
1
2
3
Terpenoids
Tocopherol
Phenlypropanoids
Simple phenols
Lignin and lignans
Alkaloids-like
Carotenoids
Flavonoids
Chalcones
Isoflavonoids
Flavonols
Flavonones
Dihydroflavonols
Anthocyanins
Glucosinolates
Cyanogenic glycosides
Sulfur containing
Betains
Wax

(b)

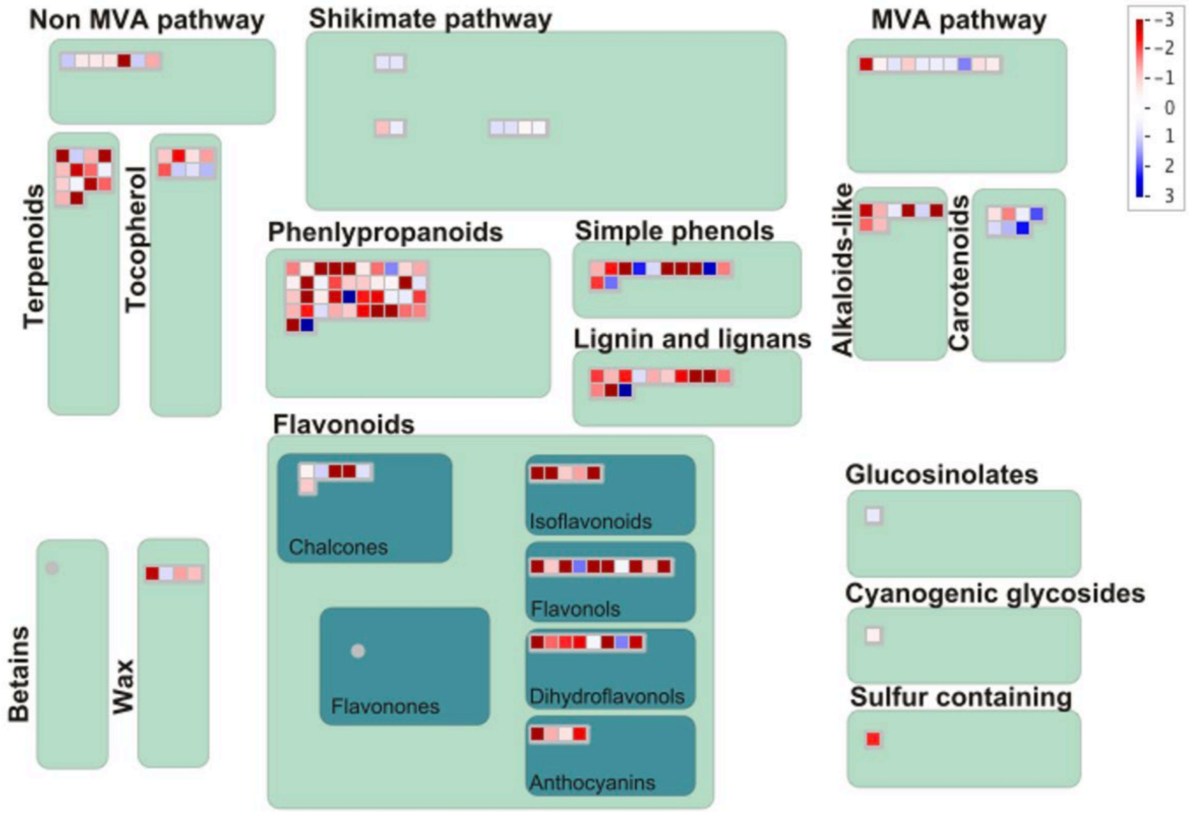
Non MVA pathway
Shikimate pathway
MVA pathway
-3
-2
-1
0
1
2
3
Terpenoids
Tocopherol
Phenlypropanoids
Simple phenols
Lignin and lignans
Alkaloids-like
Carotenoids
Flavonoids
Chalcones
Isoflavonoids
Flavonols
Flavonones
Dihydroflavonols
Anthocyanins
Betains
Wax
Glucosinolates
Cyanogenic glycosides
Sulfur containing

(c)

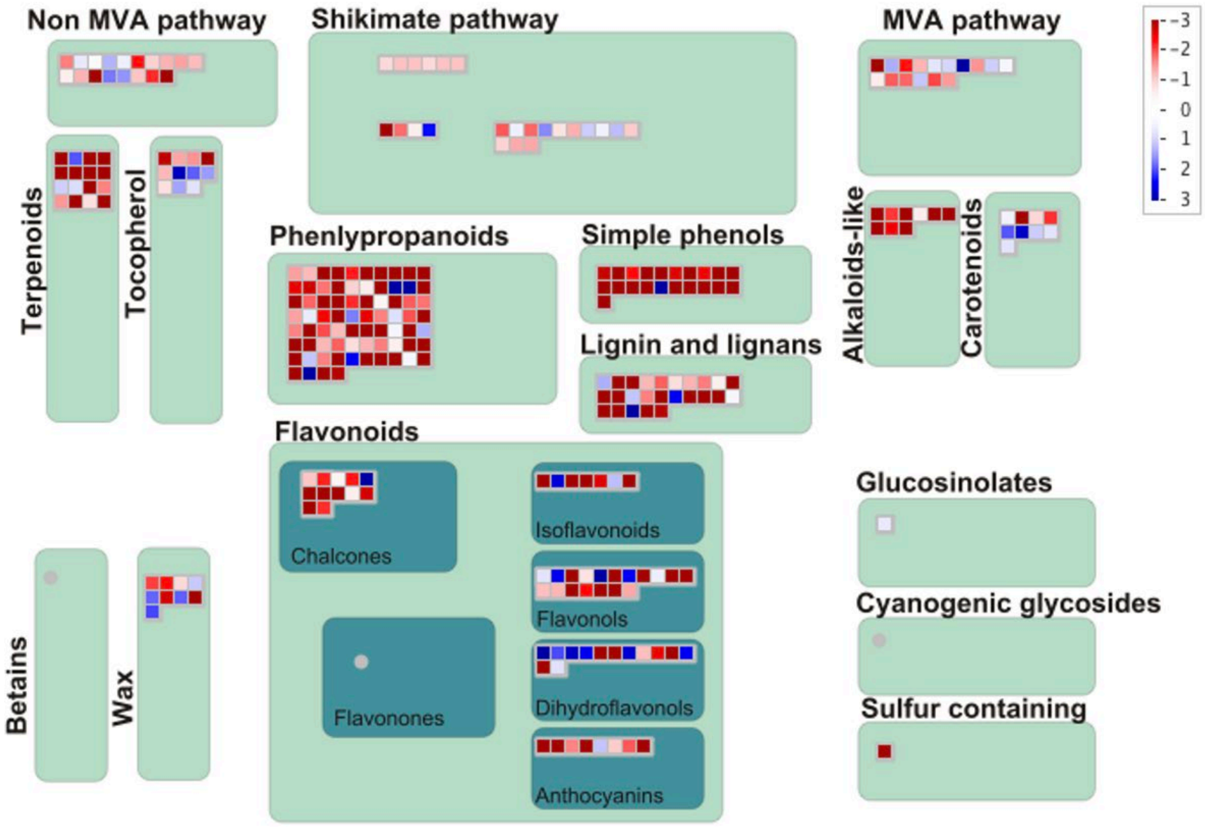


**Supplementary Figure 5. DEGs of Darij under a) flooding during germination, (b) cold stress, and (c) combined of flooding and cold stress during germination conditions associated with secondary metabolism were binned to MapMan functional categories.** Up-regulated and down-regulated transcripts are shown in blue and red, respectively.

(a)

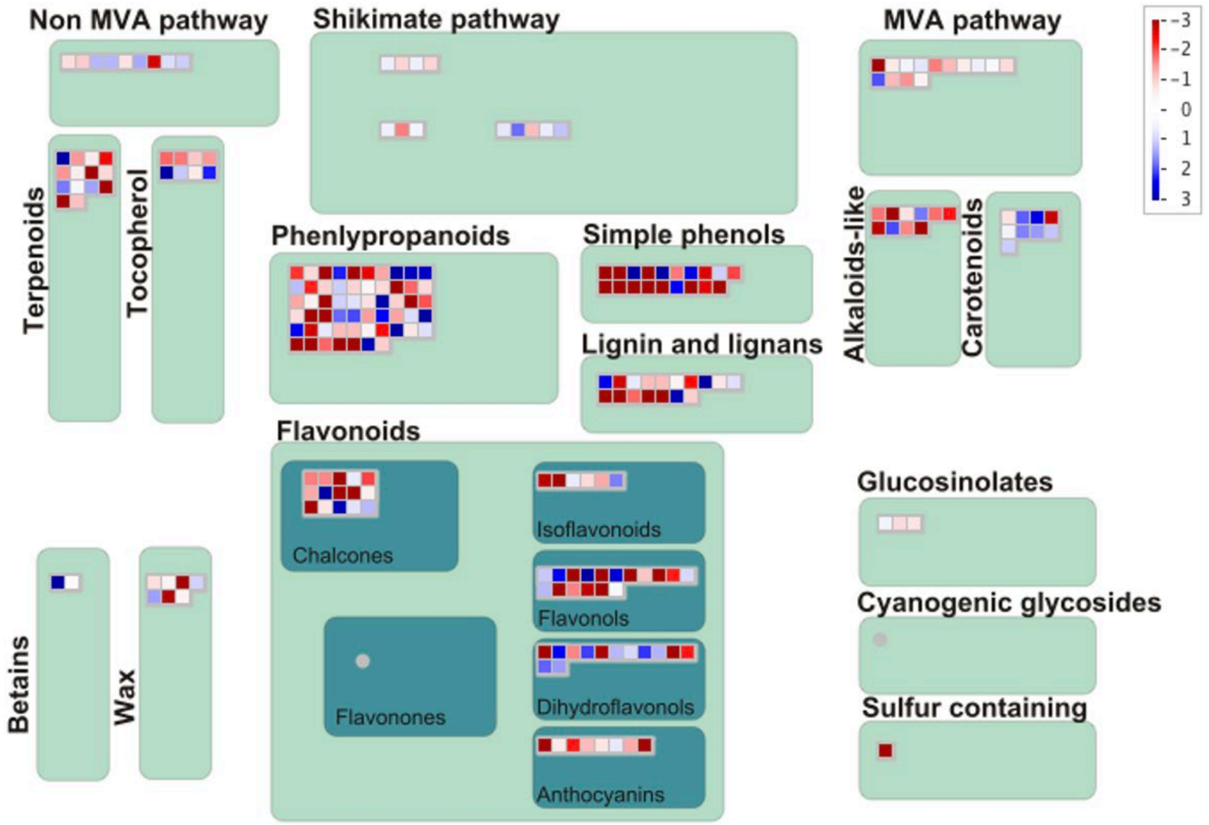

Non MVA pathway
Shikimate pathway
MVA pathway
-3
-2
-1
0
1
2
3
Terpenoids
Tocopherol
Phenlypropanoids
Simple phenols
Lignin and lignans
Alkaloids-like
Carotenoids
Flavonoids
Chalcones
Isoflavonoids
Flavonols
Flavonones
Dihydroflavonols
Anthocyanins
Betains
Wax
Glucosinolates
Cyanogenic glycosides
Sulfur containing

(b)

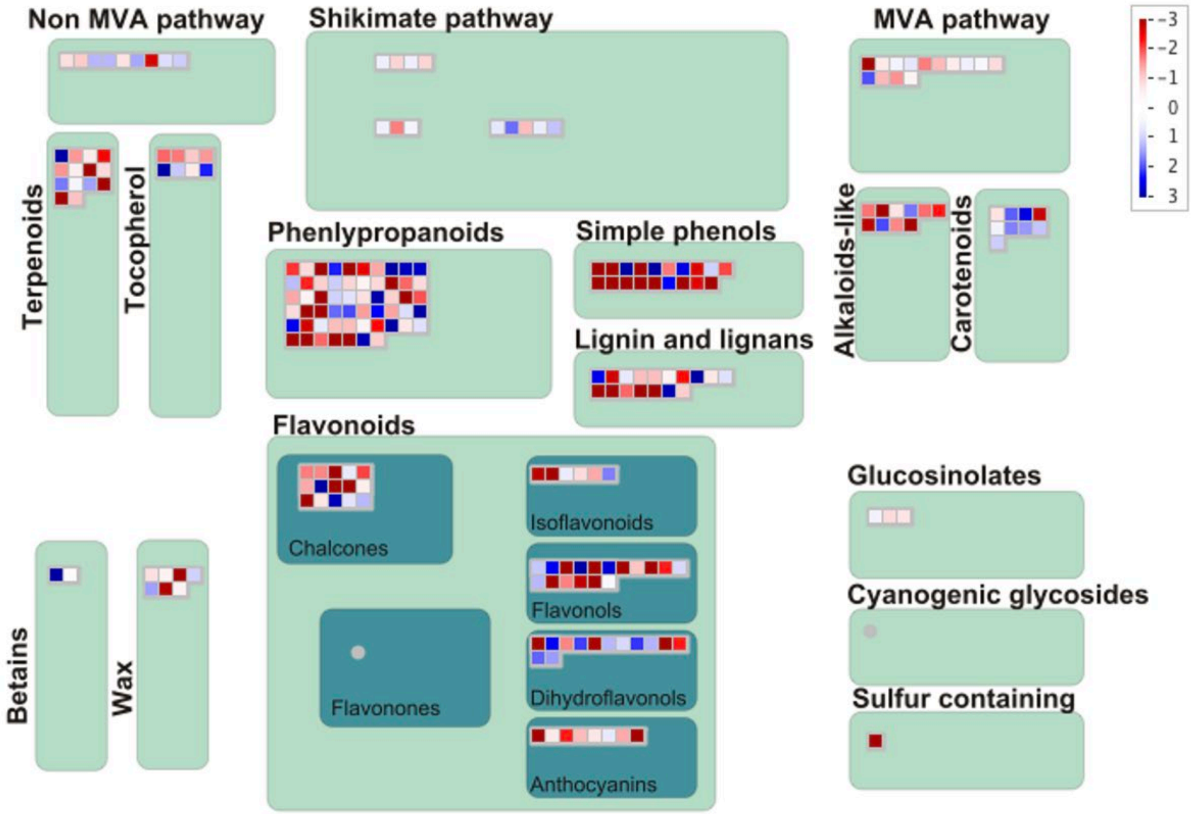
Non MVA pathway
Shikimate pathway
MVA pathway
-3
-2
-1
0
1
2
3
Terpenoids
Tocopherol
Phenlypropanoids
Simple phenols
Lignin and lignans
Alkaloids-like
Carotenoids
Flavonoids
Chalcones
Isoflavonoids
Flavonols
Flavonones
Dihydroflavonols
Anthocyanins
Betains
Wax
Glucosinolates
Cyanogenic glycosides
Sulfur containing

(c)

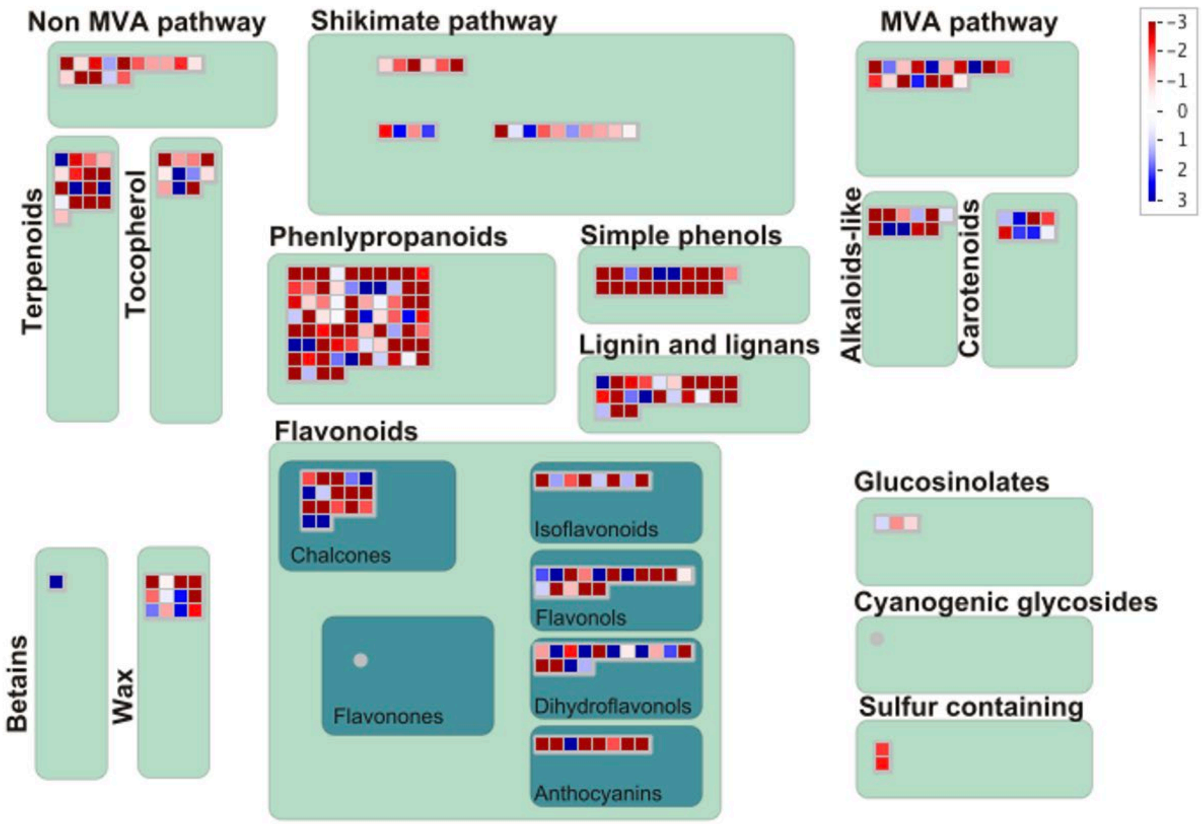


**Supplementary Figure 6. DEGs of 4610 under a) flooding during germination, (b) cold stress, and (c) combined of flooding and cold stress during germination conditions associated with secondary metabolism were binned to MapMan functional categories.** Up-regulated and down-regulated transcripts are shown in blue and red, respectively.
