# Supplementary material for: Comparative transcriptomic analysis of germinating rice seedlings to individual and combined anaerobic and cold stress
Source: BMC Genomics. 2023 Apr 6;24:185. doi: 10.1186/s12864-023-09262-z (PMC10080786; doi:10.1186/s12864-023-09262-z)
Supplement: Supplementary file 2 — Additional file 2: Supplementary Table 1. Summary of RNA seq data and sequence assembly of control samples for flooding during germination stress. Supplementary Table 2. Summary of RNA seq data and sequence assembly of samples under flooding during germination stress. Supplementary Table 3. Summary of RNA seq data and sequence assembly of control samples for cold stress. Supplementary Table 4. Summary of RNA seq data and sequence assembly of samples under cold stress. Supplementary Table 5. Summary of RNA seq data and sequence assembly of samples under combined flooding and cold stress during germination conditions. [file 12864_2023_9262_MOESM2_ESM.pdf]

Supplementary Table 1. Summary of RNA seq data and sequence assembly of control samples for flooding during germination stress

**Parameters**	**Darij (R1)**	**Darij (R2)**	**Darij (R3)**	**Darij (R4)**	**4610 (R1)**	**4610 (R2)**	**4610 (R3)**	**4610 (R4)**
Total reads	35203779	45779199	36065291	39596552	38422888	31830188	40002161	43580521
Aligned concordantly 0 times	2055103	2655340	2115356	2082729	4343032	3232098	4069481	4766254
Aligned concordantly exactly 1 time	28611944	37389333	29586704	32573082	27358263	23465355	28835485	31657075
Aligned concordantly > 1 times	4536732	5734526	4363231	4940741	6721593	5132735	7097195	7157192
Overall alignment	96.59%	96.65%	96.64%	96.78%	92.75%	93.66%	93.55%	93.30%

Supplementary Table 2. Summary of RNA seq data and sequence assembly of samples under flooding during germination stress

Parameters	Darij (R1)	Darij (R2)	Darij (R3)	Darij (R4)	4610 (R1)	4610 (R2)	4610 (R3)	4610 (R4)
Total reads	20967074	20010205	17693506	19412278	15253253	13358212	19310286	14916738
aligned concordantly 0 times	2700064	2429236	2096323	2363606	2337948	2001792	3045511	2251266
aligned concordantly exactly 1 time	14793571	14351151	12786643	13602715	9156346	8037908	11160744	8636842
aligned concordantly > 1 times	3473439	3229818	2810540	3445957	3758959	3318512	5104031	4028630
overall alignment	94.01%	94.54%	94.80%	94.16%	92.04%	91.96%	91.62%	91.93%

Supplementary Table 3. Summary of RNA seq data and sequence assembly of control samples for cold stress

Parameters	Darij (R1)	Darij (R2)	Darij (R3)	Darij (R4)	4610 (R1)	4610 (R2)	4610 (R3)	4610 (R4)
Total reads	20362711	18083685	16736485	16170585	20888662	18216918	23694743	18139624
aligned concordantly 0 times	2644232	2159764	2125647	1738368	2646276	2479763	3462627	2620915
aligned concordantly exactly 1 time	13977184	13028337	11866460	11886196	12950032	11392995	14699545	10525109
aligned concordantly > 1 times	3741295	2895584	2744378	2546021	5292354	4344160	5532571	4993600
overall alignment	93.40%	94.37%	93.60%	94.79%	92.94%	92.32%	91.94%	91.97%

Supplementary Table 4. Summary of RNA seq data and sequence assembly of samples under cold stress

Parameters	Darij (R1)	Darij (R2)	Darij (R3)	Darij (R4)	4610 (R1)	4610 (R2)	4610 (R3)	4610 (R4)
Total reads	26697720	26341887	21596650	21507424	19906704	23302620	22724476	21906681
aligned concordantly 0 times	1722596	1684399	1614216	1226206	2357162	2602197	2355298	2376885
aligned concordantly exactly 1 time	21767697	21093367	17114865	17875194	14477202	17454020	16885005	16047429
aligned concordantly > 1 times	3207427	3564121	2867569	2406024	3072340	3246403	3484173	3482367
overall alignment	96.13%	95.80%	95.68%	96.35%	92.04%	93.19%	93.29%	93%

Supplementary Table 5. Summary of RNA seq data and sequence assembly of samples under combined flooding and cold stress during germination conditions

Parameters	Darij (R1)	Darij (R2)	Darij (R3)	Darij (R4)	4610 (R1)	4610 (R2)	4610 (R3)	4610 (R4)
Total reads	23967074	22510205	26593506	29412278	25253253	23358212	29310286	24916738
aligned concordantly 0 times	2300064	2429236	2696323	2363606	2337948	2101792	2045511	2451266
aligned concordantly exactly 1 time	13563521	13425521	14654323	14502612	9054346	8027808	11150644	8762742
aligned concordantly > 1 times	3263438	3128916	2710640	3345858	3657858	3216516	5103061	4027530
overall alignment	95.01%	96.54%	95.80%	96.16%	94.04%	93.96%	92.62%	94.93%
